# Structures of the alkanesulfonate monooxygenase MsuD provide insight into C–S bond cleavage, substrate scope, and an unexpected role for the tetramer

**DOI:** 10.1016/j.jbc.2021.100823

**Published:** 2021-05-23

**Authors:** Jeremy J.M. Liew, Israa M. El Saudi, Son V. Nguyen, Denyce K. Wicht, Daniel P. Dowling

**Affiliations:** 1 Department of Chemistry, University of Massachusetts Boston, Boston, Massachusetts, USA; 2 Department of Chemistry and Biochemistry, Suffolk University, Boston, Massachusetts, USA

**Keywords:** sulfur assimilation, two-component, organosulfur, methanesulfonate, flavoenzyme, TIM-barrel, DMS, dimethylsulfide, DMSO_2_, dimethyl sulfone, DTNB, 5,5-dithio-bis-(2-nitrobenzoic acid), FADH^−^, reduced flavin adenine dinucleotide, FMN, oxidized flavin mononucleotide, FMNH^−^, reduced flavin mononucleotide, IS, insertion segment, MR, molecular replacement, MS^−^, methanesulfonate, MSI^−^, methanesulfinate, TIM, triose isomerase phosphate

## Abstract

Bacterial two-component flavin-dependent monooxygenases cleave the stable C–S bond of environmental and anthropogenic organosulfur compounds. The monooxygenase MsuD converts methanesulfonate (MS^−^) to sulfite, completing the sulfur assimilation process during sulfate starvation, but the mechanism of this conversion remains unclear. To explore the mechanism of C–S bond cleavage, we report a series of crystal structures of MsuD from *Pseudomonas fluorescens* in different liganded states. This report provides the first crystal structures of an alkanesulfonate monooxygenase with a bound flavin and alkanesulfonate, elucidating the roles of the active site lid, the protein C terminus, and an active site loop in flavin and/or alkanesulfonate binding. These structures position MS^−^ closest to the flavin N5 position, consistent with an N5-(hydro)peroxyflavin mechanism rather than a classical C4a-(hydro)peroxyflavin mechanism. A fully enclosed active site is observed in the ternary complex, mediated by interchain interaction of the C terminus at the tetramer interface. These structures identify an unexpected function of the protein C terminus in this protein family in stabilizing tetramer formation and the alkanesulfonate-binding site. Spurred by interest from the crystal structures, we conducted biochemical assays and molecular docking that redefine MsuD as a small- to medium-chain alkanesulfonate monooxygenase. Functional mutations verify the sulfonate-binding site and reveal the critical importance of the protein C terminus for monooxygenase function. These findings reveal a deeper understanding of MsuD’s functionality at the molecular level and consequently how it operates within its role as part of the sulfur assimilation pathway.

Flavin-dependent monooxygenases catalyze the monooxygenation of a wide range of substrates using dioxygen and either a reduced flavin mononucleotide (FMNH^−^) or reduced flavin adenine dinucleotide (FADH^−^), making them incredibly useful biocatalysts (1, 2, 3, 4). One family of substrates includes organosulfur compounds (5), which are of interest as the chemical properties of sulfur are crucial to many biological processes. For example, sulfur’s redox properties make the element key for electron carrier molecules such as glutathione (6) and iron-sulfur clusters (7). Biosynthetic pathways for incorporating sulfur use exogenous sulfate, which is reduced to sulfite and subsequently sulfide in the cell (Fig. 1*A*) (8). However, when deprived of typical sulfur sources, some bacteria produce sulfite from organosulfur compounds under the sulfur starvation response (9, 10, 11), using compounds including taurine (9), dibenzothiophene (12), long-chain alkanesulfonates (13), and C_1_-sulfur compounds (14) such as dimethylsulfide (DMS) (15), dimethyl sulfone (DMSO_2_) (16), and methanesulfonate (MS^−^) (17). DMSO_2_ and MS^−^ originate from the oxidation of DMS (18) in either the top layer of the ocean (19) or in aerosols (20), and deposition by rainfall (5, 21) makes DMSO_2_ and MS^−^ available for sulfur acquisition by soil-dwelling bacteria (Fig. 1*A*). In addition, the methyl groups can serve as a carbon source in methylotrophic bacteria through the serine or ribulose monophosphate pathways (22, 23). Enzymatic strategies for breaking the chemically stable C–S bond of organosulfur compounds include Rieske nonheme oxygenases (24, 25), α-ketoglutarate-dependent dioxygenases (9), and flavin-dependent monooxygenases (17, 26).

**Figure 1:**
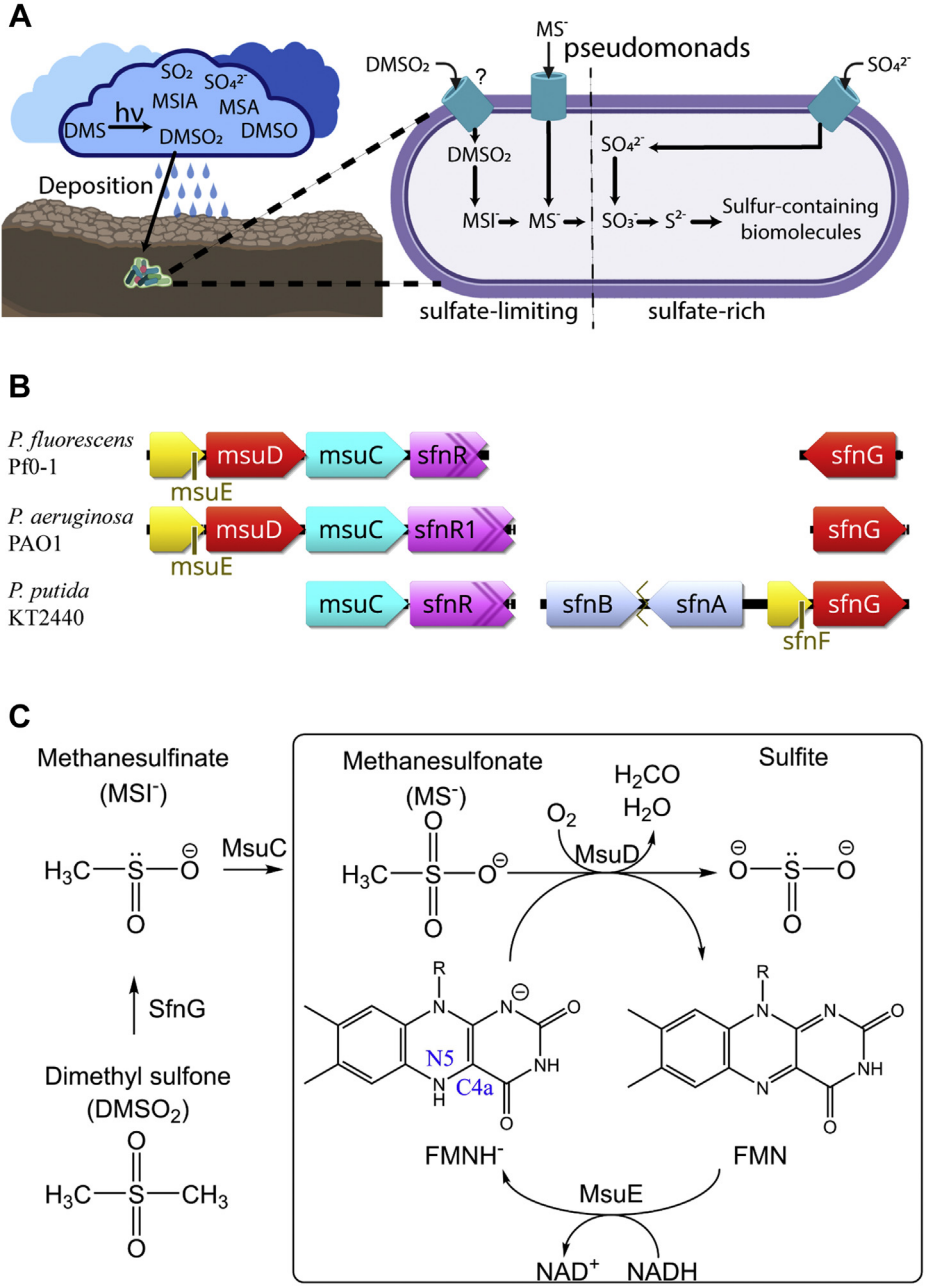
**Bacterial sulfur assimilation of dimethyl sulfone and methanesulfonate.***A*, the cycling of atmospheric sulfur is depicted. Oxidation products of dimethylsulfide (DMS) include dimethyl sulfoxide (DMSO), dimethyl sulfone (DMSO_2_), methanesulfinic acid (MSIA), methanesulfonic acid (MSA), sulfur dioxide (SO_2_), and sulfate (SO_4_^2−^). Pseudomonads can intracellularly convert DMSO_2_ into methanesulfinate (MSI^−^), methanesulfonate (MS^−^), and, finally, sulfite. Transporters for DMSO_2_ have yet to be characterized. Under sulfate-rich conditions, sulfate is reduced to sulfite and subsequently sulfide (S^2−^) for biosynthetic reactions. Representation created with BioRender.com. *B*, representative gene clusters for utilization of DMSO_2_ from *P. fluorescens* Pf0-1, *P. aeruginosa* PAO1, and *P. putida* KT2440 include three monooxygenases, *sfnG*, *msuC*, and *msuD*, and the oxidoreductases *msuE or sfnF*. Monooxygenases that break C–S bonds are in *red*, monooxygenases that form S–O bonds are in *cyan*, NADH:FMN oxidoreductases are in *yellow*, transcriptional regulators are in *pink*, and proteins of unknown function are in *light blue*. Initial studies of the *sfn* operon were reported for the genetically intractable *P. putida* DS1 (16, 29); therefore, *P. putida* KT2440 was used for alignment. The *sfn* operon in *P. putida* contains the uncharacterized *sfnAB* pair (79), which is separated by predicted ABC transporters and a hypothetical protein (represented as a *jagged line*); however, *sfnG* is a stand-alone gene within *P. aeruginosa* PAO1 (80) and *P. fluorescens* Pf0-1. The *msu* operon groups with *sfnR*, or sfnR1 and sfnR2 in *P. aeruginosa* (sfnR2 omitted from alignment) (80). Gene information is in Table S1. *C*, the sulfur assimilation pathway of *P. fluorescens* converts DMSO_2_ into sulfite. The NADH:FMN oxidoreductase MsuE is required to produce FMNH^−^ for SfnG, MsuC, and MsuD; however, MsuE is shown only for the MsuD reaction for clarity.

In pseudomonads, *msu* and *sfn* genes contain the flavin-dependent monooxygenases SfnG, MsuC, and MsuD that convert DMSO_2_ to sulfite (Fig. 1*B*). SfnG converts DMSO_2_ to methanesulf**in**ate (MSI^−^) (27), MsuC oxidizes MSI^−^ to methanesulf**on**ate (MS^−^) (28), and MsuD catalyzes the conversion of MS^−^ to sulfite (Fig. 1*C*) (17). Together SfnG and MsuD are responsible for sequential cleavage of the two C–S bonds of DMSO_2_, and each methyl group is presumed to be oxidized to formaldehyde. The enzymes SfnG, MsuC, and MsuD are members of a small subset of flavin-dependent monooxygenases that are characterized by their use of reduced flavin as a cosubstrate rather than a cofactor. Termed two-component flavin-dependent monooxygenases, members of this family lack an NAD(P)H-binding site and therefore require a separate reduced NAD(P)H:oxidized flavin mononucleotide (FMN) oxidoreductase to provide the FMNH^−^ cosubstrate. MsuE was identified within the *msu* operon of *Pseudomonas fluorescens* and *Pseudomonas aeruginosa* as the likely NADH:FMN oxidoreductase for MsuC (28) and MsuD (17), and SfnF was identified in the *sfn* operon of *Pseudomonas putida* (29) (Fig. 1*B*). Structurally characterized two-component flavin-dependent monooxygenases that catalyze C–S bond cleavage include CmoJ, DmoA, BdsA, and SsuD, which adopt a (β/α)_8_ triose isomerase phosphate (TIM) barrel fold and are denoted as group C two-component flavin-dependent monooxygenases (1). This is separate from MsuC, a group D flavin-dependent monooxygenase that instead exhibits the acyl-CoA dehydrogenase fold and catalyzes formation of a S–O bond. The group C monooxygenase DmoA functions on dimethylsulfide (30), CmoJ is involved in the cysteine salvage pathway (31), and BdsA is part of the 4S utilization pathway to convert dibenzothiophene to sulfite (32). However, MsuD is most closely related to *Escherichia coli* alkanesulfonate monooxygenase SsuD with 67% sequence identity. SsuD catalyzes C–S bond cleavage of C_2_-C_10_ alkyl-substituted sulfonates, in addition to alkyl chains with bulkier chemical groups and larger buffer molecules (26), whereas MsuD is reportedly most active with a C_1_ sulfonate (17). To date, no known alkanesulfonate monooxygenase structure has been solved with flavin or an alkanesulfonate bound, and only the structure of *E. coli* SsuD without ligands has been reported. As such, the disorder of the SsuD active site has hindered our understanding of the vital alkanesulfonate monooxygenase reaction (33).

The overall monooxygenase reaction was originally thought to advance through a C4a-(hydro)peroxyflavin intermediate (3), but recent structural, computational, and biochemical studies of two monooxygenases, EncM from enterocin biosynthesis (34) and RutA from uracil degradation (35), support the use of either an N5-(hydro)peroxyflavin or flavin-N5-oxide. Although EncM is structurally similar to flavin oxidases/dehydrogenases (36) and RutA exhibits a TIM barrel fold (37), their structures identified that dioxygen and the substrate bind on opposite faces of the flavin, and a conserved dioxygen reactivity motif (F/L-T/S-NxV-A-F/L/Y, where x is a branched chain amino acid) was identified within RutA and a subset of group C monooxygenases, including CmoJ, DmoA, and DszA (catalyzes the same reaction as BdsA). Matthews *et al.* (35) investigated RutA *via* computational studies that support the thermodynamic possibility of inversion at the N5 position of the flavin, which would move the (hydro)peroxy group toward the substrate for catalysis to proceed. RutA and MsuD share only 25% sequence identity, and the dioxygen reactivity motif of RutA is not fully conserved in MsuD, SsuD, or SfnG; therefore, it is unclear if a similar N5-(hydro)peroxyflavin could be employed in enzymes that facilitate C–S bond cleavage. Observing the ordered active site would help elucidate this question. Likewise, a completely ordered model could help clarify regions of disorder that exist in RutA and other group C monooxygenases such as the active site lid and seemingly functionless C terminus.

Here we report the first crystal structures and biochemical activity studies of *P. fluorescens* MsuD, which adopts a group C (β/α)_8_ TIM barrel fold. Structural experiments entailing soaks and cocrystallization of MsuD with FMN and MS^−^ enabled us to obtain the first ever crystallographic snapshots of an alkanesulfonate monooxygenase in different complexed states with its substrates. Because the FMNH^−^ cosubstrate is aerobically sensitive (38, 39), we have employed oxidized FMN, a product of the monooxygenase, to obtain crystal structures that detail the flavin-binding site. We report the structures of unliganded MsuD, MsuD in complex with FMN, and MsuD in complex with both FMN and MS^−^. The 2.4-Å resolution structure of MsuD with flavin and MS^−^ has enabled us to revisit the chemical mechanism to assess if C–S cleavage likely advances through the C4a or N5-(hydro)peroxyflavin intermediates, as well as put into context many of the biochemical experiments that have been difficult to rationalize without an experimentally determined binding mode for the flavin and alkanesulfonate moieties. Our studies further have led us to redetermine the substrate scope for MsuD. The resulting structures reveal an exquisite molecular connection between the flavin- and MS^−^-binding sites, the active site lid, and the protein C terminus, providing an unrealized function for the C-terminal extension that may be used in other group C flavin monooxygenases.

## Results

### Overall structure of MsuD from *P. fluorescens*

**Figure 2:**
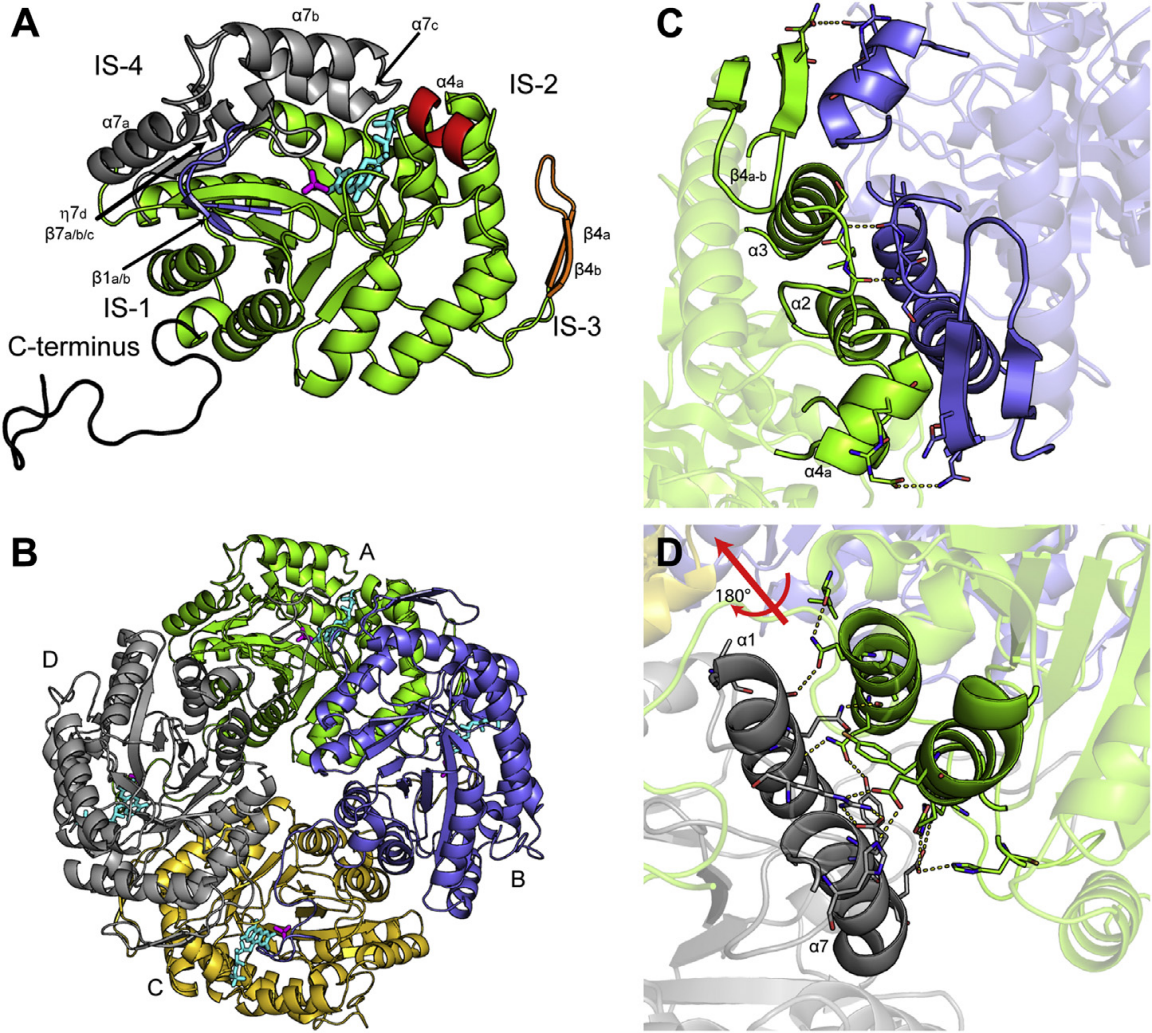
**Structure of MsuD from *P. fluorescens*.***A*, MsuD adopts a classic (β/α)_8_ barrel (*green*) with four insertion regions, labeled IS-1 (*blue*), IS-2 (*red*), IS-3 (*orange*), and IS-4 (*gray*). The extended protein C terminus is colored *black*. FMN (*cyan*) and MS^−^ (*magenta*) are displayed as *sticks*. *B*, MsuD adopts a dimer-of-dimers between chains A and B and chains C and D. Protein chains are labeled A–D and colored *green*, *blue*, *yellow*, and *gray*, respectively. *C*, the dimer and *D*, tetramer interfaces are displayed with labels for only half of the symmetrical interfaces for clarity. Hydrogen bonding networks are drawn as *yellow dashed lines*.

The 2.4-Å resolution crystal structure of MsuD in complex with FMN and MS^−^ (ternary-MsuD) was obtained by cocrystallization with greater than 5-fold molar excess of both ligands. MsuD adopts a classic (β/α)_8_ TIM barrel fold (Fig. 2*A*) (40), with four insertion segments (ISs) to the (β/α)_8_ core of MsuD: IS-1 includes two β-strands (labeled β_1a/b_); IS-2 includes an α-helix (labeled α_4a_); IS-3 includes a β-hairpin (labeled β_4a/b_); and IS-4 includes three α-helices (labeled α_7a/b/c_), a 3_10_ helix (labeled η7_d_), and three short β-strands (labeled β_7a/b/c_). In addition, MsuD contains an extended protein C terminus. All but the last four residues of the protein C terminus were able to be placed into electron density along with the ligands FMN and MS^−^.

Ternary-MsuD crystallized in the space group *P*6_1_ with four MsuD chains per asymmetric unit, arranged as a dimer-of-dimers (Fig. 2*B*). The dimer and tetramer interfaces bury approximately 1960 and 2940 Å^2^ per molecule, respectively, determined by the PISA server (41). The buried surface area is consistent with size exclusion data that support MsuD is a tetramer (Fig. S1*A*). The dimer interface consists of portions of the (β/α)_8_ core, IS-2, and IS-3 (Fig. 2*C*), where the β_4a/b_-hairpin packs against α_4a_ of the dimer molecule. The main tetramer interface is made up of components from the (β/α)_8_ core, where α_1_ and its preceding loop as well as α_8_ combine with a symmetry-related molecule to generate a four-helix bundle (Fig. 2*D*). The ordering of the C terminus constitutes approximately 1650 Å^2^ of the tetramer interface, making the tetramer interface the largest interface between monomeric subunits; however, as will be discussed below, the C terminus appears to be a mobile element that is related to substrate binding. It is intriguing that truncation of the last 16 residues from the C terminus affects migration of the protein by gel filtration (Fig. S1*B*), causing MsuD to behave as a dimer.

**Figure 3:**
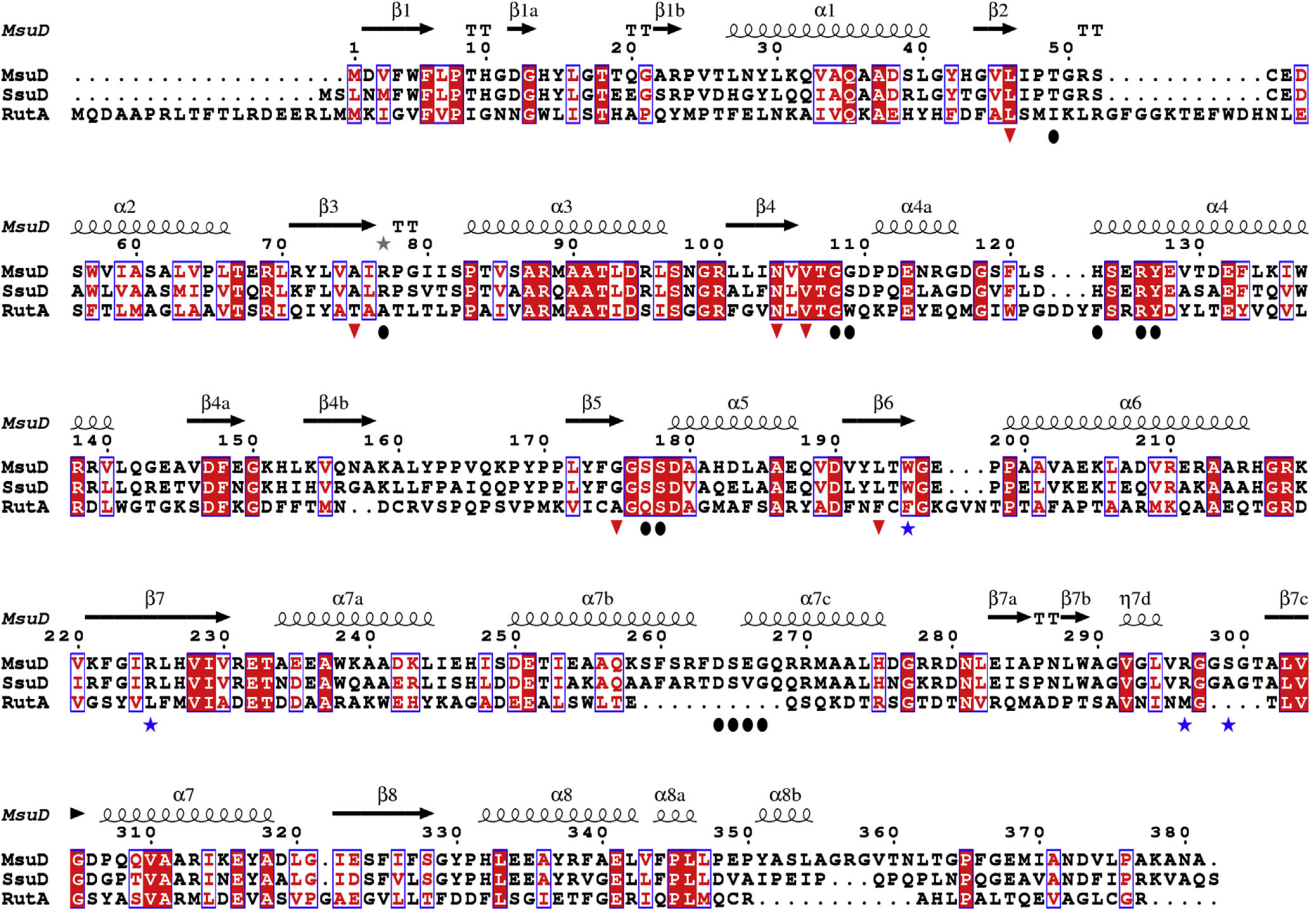
**Sequence alignment of *P. fluorescens* MsuD with *E. coli* SsuD and RutA.***Circles* represent residues involved in FMN binding, *red triangles* are part of the proposed dioxygen binding motif, and *blue stars* are residues directly involved in MS^−^ binding within MsuD. An extended sequence alignment with structural homologs is included in Fig. S2. *Blue boxes* indicate conserved and similar residues, with strictly conserved residues highlighted in *red* and similar residues in *red text*. Secondary structural elements are shown for MsuD.

As was predicted, the closest reported structure to MsuD is *E. coli* SsuD (42), which aligns with an RMSD of 1.3 Å for 330 cα atoms (43). Related structures with less than 32% sequence identity are included in Table S2, and sequence alignment of MsuD with SsuD and a more distantly related homolog representative, RutA, is shown in Figure 3. Like MsuD, both SsuD and BdsA crystallize as homotetramers (Fig. S3), whereas the other structural homologs are reported homodimers. The observed protein–protein interfaces are highly conserved with SsuD (33); however, there is less than 15% conservation of interfacing residues with other structurally characterized group C monooxygenases. Therefore, this enzyme family can accommodate greatly divergent sequences that still preserve the overall quaternary structure.

### The active site of MsuD is solvent exposed without FMN bound

**Figure 4:**
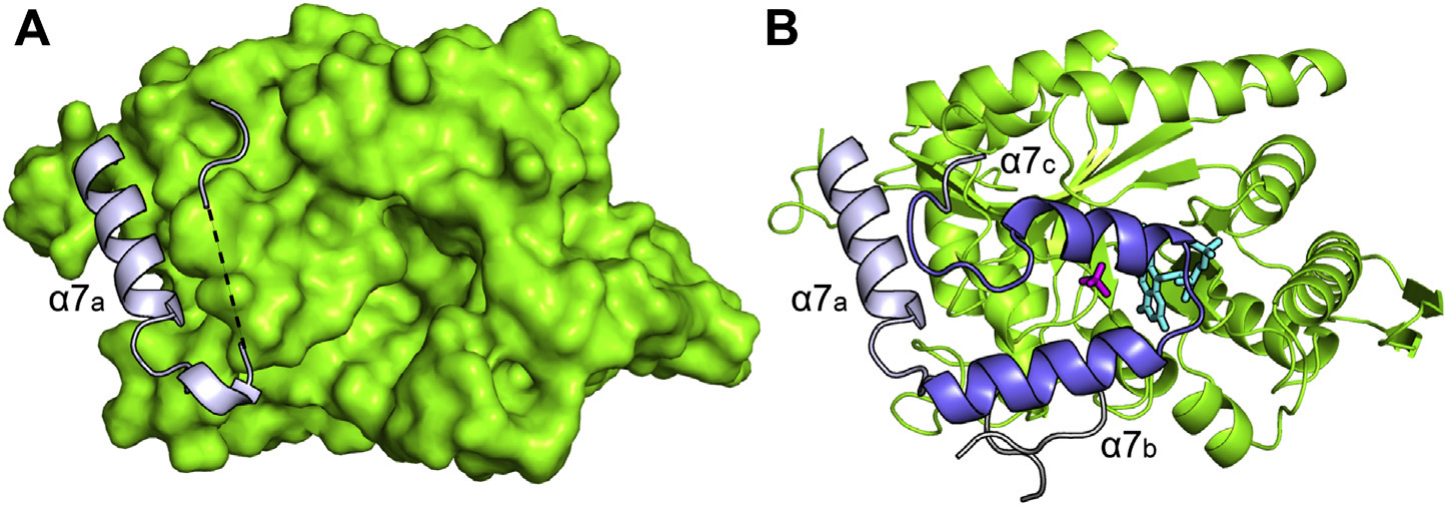
**The lid of MsuD is important for active site enclosure.***A*, the active site lid is disordered in the unliganded MsuD structure, which is displayed as a *green* surface with portions flanking the lid region as *ribbon* (*blue-white*). The disordered region is represented as a *dashed black line*. *B*, the ternary-MsuD structure contains an ordered lid (*blue*) and an ordered C terminus from a separate protein molecule (*gray*). FMN (*cyan*) and MS^−^ (*magenta*) are displayed as *sticks*.

In the absence of ligands, MsuD crystallizes in space group *P*2_1_ with two MsuD tetramers (chains A/B/C/D and E/F/G/H) per asymmetric unit. The 2.8-Å resolution crystal structure of unliganded MsuD provides a snapshot of the enzyme prior to FMNH^−^, MS^−^, and O_2_ binding. The two tetramers align with an overall RMSD of 0.34 Å for 1188 cα atoms, indicating their structures are largely identical. Of interest, residues in all chains within IS-4 (between α_7a_ and β_7a_) and from L354 to the C terminus lack any electron density, preventing their modeling and resulting in a very open structure (Fig. 4*A*). In each chain of ternary-MsuD, α_7b_ and α_7c_ from IS-4, as well as the protein C terminus of an adjacent chain, are ordered and observed to enclose the C-face of the (β/α)_8_ barrel, covering FMN and MS^−^ and blocking solvent access to the active site (Fig. 4*B* and electron density is shown in Fig. S4, *A*–*C*). The region spanning residues 250 to 282 in SsuD is similarly disordered (33) and reported as a “mobile loop region” (44). We will refer to the α_7b_–α_7c_ region (D250-L282) as a “lid.” In ternary-MsuD, we observe that the two lid helices pack antiparallel to each other and are connected by a three-residue linker (F263-S265). Both hydrophobic and polar interactions contribute to stabilizing the antiparallel positioning of α_7b_ and α_7c_, which are splayed apart at an angle of 32.7°.

To further explore the results of ligand binding on MsuD, FMN alone or FMN with MS^−^ was soaked into crystals of unliganded MsuD using excess concentrations of each ligand. Ligand binding and protein ordering results are summarized in Table S3. Datasets for crystals soaked with FMN were prepared in two ways: an overnight soak with >1 mM FMN (binary-soak MsuD) and an overnight soak titrated with a low concentration of FMN until crystals became pale yellow (binary-titrated MsuD). Both crystals contained 2 mM FMN in their cryoprotectants, yielding datasets of approximately 2.8-Å resolutions. In both structures, six FMNs were placed but distributed differently between the two tetramers. Binary-soak MsuD contained FMN in chains A/B/C/D of the first tetramer and chains E/G of the second tetramer, whereas binary-titrated MsuD contained FMN in chains A/C/D and E/G/H. Chains A, C, and E contain the strongest electron density for FMN in both structures (Fig. S4, *D* and *E*). FMN binding led to ordering of the active site lid only when FMN bound with nearly full occupancy, and the B-factor values for portions of these lids are nearly double the average protein B factor, indicative of lid mobility within the crystal. Only one or three C termini were observed in both FMN-soaked structures: chain H in binary-soak MsuD extends into the active site of FMN-bound chain E, and chains B, D, and H in binary-titrated MsuD extend into the FMN-bound active sites of chains C, A, and E, respectively. For each active site that has an inserted C-terminal tail, electron density is within the sulfonate-binding site that best refines as a succinate from the crystallization condition, likely serving as a mimic of MS^−^.

MsuD soaked with FMN and MS^−^ (ternary-soak MsuD) was solved to 2.7-Å resolution, and four FMN with MS^−^ pairs were placed within all protomers of the first tetramer (chains A/B/C/D) (electron density displayed in Fig. S4*F*). Within the second tetramer, three FMN molecules were placed within chains E/G/H, and MS^−^ could only be placed in chain E. Ligand binding similarly led to ordering of the active site lid with FMN bound at nearly full occupancies; the lid was observed in chains A/B/C/D of the first tetramer and chain E of the second tetramer. Chains D, G, and H have varying degrees of disorder for the lid region (Table S3). The C terminus is only observed for chain H, where it extends into the active site of the FMN-bound chain E. Of importance, weaker electron density for FMN and MS^−^ were observed in protomers containing a disordered lid region.

Taken together, ordering of the lid and the C terminus is linked to binding of FMN at near full occupancy. FMN binding appears to favor binding fully to one molecule of each homodimer related by C_2_ symmetry (chains A/C and E/G in these structures) prior to binding in the remaining tetramer active sites. Binding of a succinate within the sulfonate-binding site is observed only with an ordered interaction of both the lid of the same protomer and the C terminus from an adjacent monomer, whereas MS^−^ requires ordering of the lid but can bind without an ordered C terminus. Variable numbers of ordered C termini were observed between the soaked structures, indicative of the higher mobility of the C terminus. We also cannot rule out that ordering of the lid and C terminus within an entire tetramer may be perturbed by the crystal lattice, which would explain why cocrystallization of ternary-MsuD yielded a tetramer with four ordered lids and four ordered C termini.

### FMN binding to MsuD

**Figure 5:**
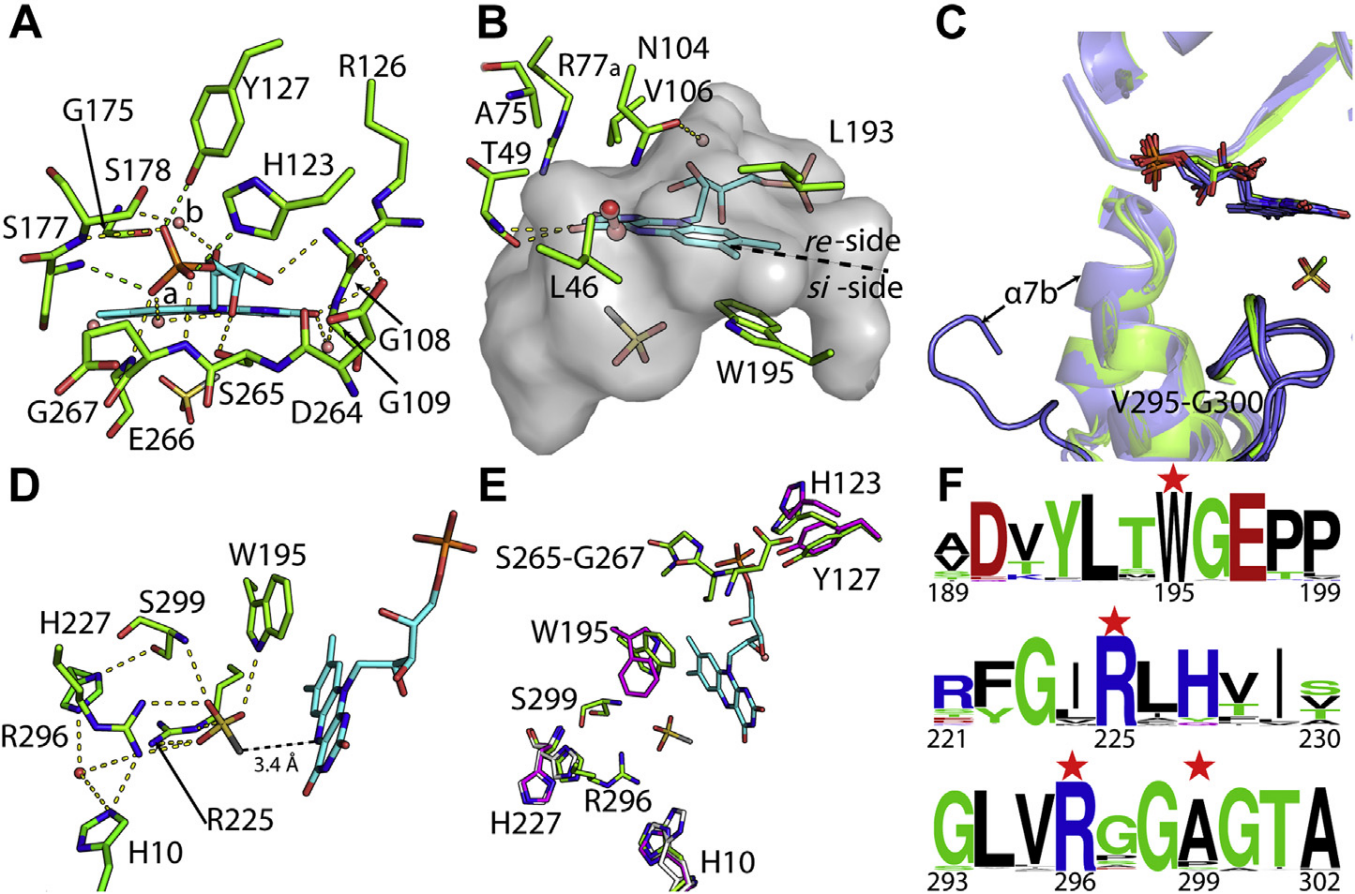
**Residues involved in flavin and MS**^−^**binding and a potential dioxygen-binding site.***A*, extensive polar contacts are made to the phosphate head and hydroxyls of the ribityl arm. *B*, Isoalloxazine interacting residues are shown. Residues of the dioxygen reactivity motif generate a partial pocket at the *re*-face of FMN where dioxygen is proposed to bind. Dioxygen is modeled in *red sphere and stick* representation from alignment of the FMN isoalloxazine ring of MsuD with the FMN of O_2_-bound RutA (Protein Data Bank ID 6SGG). The active site cavity is displayed as a *gray* surface. *C*, orientations of the ribityl arm and the loop of V295-G300 show alternate conformations within different chains of binary-soak MsuD (*blue*) in comparison with ternary-MsuD (*green*). An alternative orientation for α7_b_ of the lid region in chain H is observed to direct the active site lid away from the FMN-binding site. *D*, MS^−^ binding positions the methyl group of MS^−^ approximately 3.4 Å from the N5 of FMN. *E*, an overlay of ternary-MsuD (chain A), binary-MsuD (FMN-bound chain A and unliganded chain H), and unliganded MsuD (chain A) shows residues that have alternate conformations when FMN is not present. Colors are as follows: ternary-MsuD carbons, *green*; binary-MsuD carbons, *white*; unliganded-MsuD carbons, *magenta*; FMN carbons, *cyan*; MS^−^ carbon, *gray*; oxygen, *red*; nitrogen, *blue*; sulfur, *yellow*; phosphorus, *orange*; and waters, *pink*. *F*, sequence logos are shown for the sulfonate-binding motif of flavin-dependent alkanesulfonate monooxygenases with *red stars* for interacting residues.

Chain A of ternary-MsuD was used to analyze FMN binding as it is the most ordered chain of the tetramer and displays the lowest average B factor value. FMN binds at the C-terminal face of the TIM barrel and is positioned to engage in several conserved hydrogen-bonding and van der Waals interactions (Fig. 5*A* and Fig. S5). The phosphoryl moiety of FMN engages in seven or eight hydrogen-bonding interactions with the side chains of H123, Y127, and S178; the backbone NH groups of S177, S178, E266, and G267; and an ordered water molecule observed within chains A, B, and C (labeled “a”). The ribityl 3′-OH is approximately 3.5 Å from the backbone NH groups of G108 and G109, and the 4′-OH interacts with the hydroxyl of S265 and water “a.” The FMN 2′-OH is positioned at the *re*-face of the isoalloxazine ring where it interacts with the backbone C=O of G175 and a crystallographic water (labeled “b”). Of interest, S265, E266, and G267 are located within the active site lid, and D264 of the same region is observed to interact with a conserved arginine (R126) from near the phosphoryl-binding site. The observed binding mode of FMN is expected to be similar for FMNH^−^ and is consistent with mutagenesis studies in the homolog BdsA, where mutations to residues corresponding to H123, R126, Y127, and S177 reduced the turnover rate by 45% to 95% (45).

The isoalloxazine ring of FMN binds with the O4 and N3 of its pyrimidine moiety positioned within hydrogen bonding distance to the backbone NH and C=O of T49 and its O2 near the guanidinium of R77 (Fig. 5*B*); however, R77 is more disordered and observed in multiple orientations within chains A, B, and D. A π-stacking interaction is observed on the *si*-face of FMN with W195 (which adopts a different conformation with high B factor values in structures without FMN). The corresponding aromatic residue in unliganded homologous structures is directed away from the flavin-binding site (Fig. S6*A*). A pocket is formed by L46, T49, A75, N104, V106, and L193 on the *re*-face of the isoalloxazine ring where dioxygen likely binds (Fig. 5*B*), and comparatively no protein atoms are located within 5.4 Å of the C4a or N5 flavin positions that could stabilize dioxygen binding at the *si*-face. Indeed, dioxygen-binding studies within RutA (35) have identified that the *re*-face of the isoalloxazine ring is where dioxygen is expected to bind within several group C flavin-dependent monooxygenases and be positioned to react with the N5 as opposed to the C4a of flavin. The residues within MsuD that form the pocket share 50% sequence identity with RutA and are largely consistent in atomic coordinates; however, N104 and L46 are positioned closer to N5 of FMN than in RutA and result in a smaller binding pocket for dioxygen. An overlay of the FMN isoalloxazine rings from dioxygen-bound RutA and MsuD reveals the dioxygen site is too small. This binding mode is likely a result of having the product, oxidized FMN, bound. No other pocket is observed that could facilitate dioxygen binding. Therefore, the likely dioxygen-binding site in MsuD is on the *re*-face of FMN at the dioxygen reactivity motif, and formation of an N5-peroxyflavin would be consistent with the positioning of the dioxygen pocket closest to the N5 position on the *re*-face of the flavin and the need for inversion to position the reactive peroxy group on the *si*-face, within reaction distance of MS^−^ (detailed below).

Although group C flavin-dependent monooxygenases contain highly conserved flavin-binding pockets, the ribityl moiety of FMN is observed in different orientations. MsuD binds FMN with two ribityl torsion angles less than 80° and the 3′-OH 2.8 Å from N1 of the isoalloxazine ring, most closely resembling the structure of LadA (Fig. S6*B* and Table S4) (46). Of interest, in MsuD the crystallographic water near the FMN 2′-OH group is positioned to interact with N104 from the expected dioxygen-binding site (Fig. 5*B*). Therefore, the configuration of the FMN ribityl moiety may communicate to the proposed dioxygen-binding site by ordering N104 through a water-mediated hydrogen bond. In support of this hypothesis, the ribityl moiety of FMN observed within binary-soaked MsuD has less well-defined electron density than in ternary-MsuD (Fig. 5*C* and Fig. S4, *C* and *D*), particularly in monomers that have FMN partially bound but without an ordered lid. It is intriguing that alternate configurations for the loop V295-G300 near MS^−^ are observed and a second conformation is observed for residues S249–R272 (α7_b_) in the lid of chain H. In this alternate lid conformation, the lid is directed away from the active site and the residues no longer adopt an ordered α-helical structure.

### Methanesulfonate binding to MsuD

Within the structure of ternary-MsuD, a tetrahedral-shaped electron density peak is observed at the *si*-face of FMN in each molecule of the tetramer. Placement of MS^−^ within the electron density refines nicely with B factors that are comparable with the FMN and nearby protein residues. The binding mode of MS^−^ positions its oxygen atoms within an extensive hydrogen-bonding network with the guanidiniums of R296 and R225, the indole NH of W195, and the backbone NH of S299 (Fig. 5*D*), resulting in placement of the methyl group 3.4 and 3.9 Å from the N5 and C4a atoms of FMN (respective average distances for the tetramer), whereas the sulfur is approximately 4.4 Å away from the N5. Therefore, the observed binding mode supports the methyl of MS^−^ as the site of oxygenation, and the closer distance to the N5 of FMN is consistent with use of an N5-peroxyflavin intermediate (35). To further validate the sulfonate-binding site, the R225A, R296A, and W195A variants of MsuD were generated (Fig. S7) and assayed in a coupled reaction with MsuE for conversion of MS^−^ to sulfite. In comparison with wildtype MsuD, which generated 28.5 ± 1.1 μM sulfite within a 5-min reaction, no measurable sulfite was detected for R225A, R296A, or W195A.

Apart from R225, many of the residues that interact with MS^−^ are observed in alternative conformations or are completely disordered in unliganded MsuD (Fig. 5*E*), which supports binding of flavin prior to MS^−^. The W195 interaction uses π-stacking to orient the indole NH group of FMN. The positioning of R225, R296, and S299 in ternary-MsuD is stabilized by two histidine residues. H10 adopts a nonstandard rotamer to interact with R296, and H227 packs edge-to-face against the guanidinium of R296 and is within hydrogen bonding distance of S299 and an ordered water molecule (Fig. 5*D*). A mutation in SsuD of the residues analogous to H10 and H227 showed little or no change in activity relative to wildtype, supporting a role for both H10 and H227 as secondary shell interactions to the MS^−^-binding site (47). Both R296 and S299 are located within the V295-G300 loop connecting η_7d_ and β_7c_ after the lid region, and these residues are completely disordered in unliganded MsuD or are observed in alternate conformations with FMN absent or at partial occupancy (Fig. 5, *C*–*E*). As this loop contains half of the residues directly interacting with MS^−^, it is being termed the sulfonate-binding loop, with the consensus sequence RGGA identified from an alignment of 6428 sequences demonstrating between 30% and 40% sequence identity with MsuD (Fig. 5*F*). Indeed, the MsuD R296A variant reported here demonstrates no sulfite production, and mutations in SsuD of the arginine analogous to R296 to alanine or cysteine similarly resulted in a loss of enzyme function, with a lysine substitution yielding a 30-fold decrease in catalytic efficiency (44). Our structure of ternary-MsuD therefore has identified the sulfonate-binding motif of group C flavin-dependent alkanesulfonate monooxygenases to include W195 and R225 of the (β/α)_8_ core and R296 and A299 (S299 in MsuD) from the sulfonate-binding loop.

### An identified function of the protein C terminus in flavin monooxygenases

**Figure 6:**
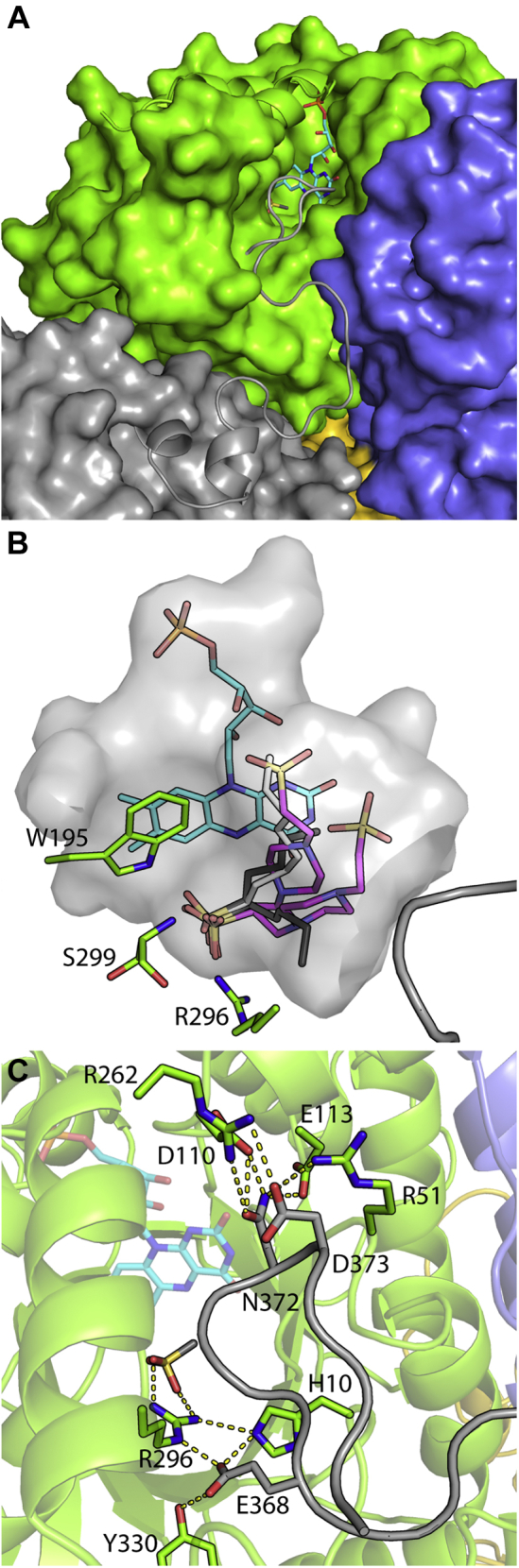
**C terminus interactions within ternary-MsuD.***A*, the active site of MsuD chains A and B are displayed as *green* and *blue* surfaces with *ribbons* corresponding to the active site lid and the C terminus of chain D (*gray*). An identical interaction is observed for all chains in ternary-MsuD. *B*, example poses of docked C5 (*gray*) and C8 (*light gray*) sulfonates, and the buffer Pipes (*magenta*), position the sulfonate moiety similarly to MS^−^, but with variable conformations of the substrate. The active site cavity is displayed as a *gray* surface with a gap where the C terminus encloses the active site. R296 is omitted for clarity. Hepes and Mops docking results had similar placements of the sulfonate moiety but are omitted for clarity as both represent smaller buffer molecules compared to Pipes. *C*, polar interactions of the C terminus are shown.

Available structures of group C FMN-dependent monooxygenases all contain a relatively open active site, despite positioning of the lid region when flavin binds.These proteins contain a long, disordered protein C terminus of unidentified function (30, 33, 37, 45, 48). To our surprise, the dataset of ternary-MsuD contained contiguous electron density for residues A355-A377 of the C terminus. The C terminus includes two short helices, a 3_10_ helix from F344-L347 and an α-helix from Y351-A355, followed by a long meandering loop that extends from each protein molecule, inserting itself within the active site of the adjacent molecule at the tetramer interface (Figs. 2*B* and 6*A*). Solvent-exposed portions of this tail display higher B factors and weak electron density; however, residues that interact with the adjacent protein molecule are well ordered with electron density for side chains.

When both the lid and C terminus are ordered and bound in ternary-MsuD, the active site appears completely enclosed from bulk solvent. The apparent volume is larger than MS^−^, consistent with previously observed activity against larger sulfonate substrates. Therefore, molecular docking of substrates ranging in size from pentanesulfonate to Pipes was explored. Docking returned possible poses with the sulfonate moiety in a similar orientation as observed for MS^−^, but with variable positioning of alkyl groups (Fig. 6*B*).

### MsuD from *P. fluorescens* is an alkanesulfonate monooxygenase

To explore if the docked poses for larger alkanesulfonates could be competent for catalysis, recombinantly expressed MsuD from *P. fluorescens* was assayed with C_1_, C_5_, and C_8_ sulfonates and the buffer molecules Hepes, Pipes, and Mops in a coupled reaction with MsuE. MsuD converted MS^−^ to sulfite with an activity of 26 ± 2 μmol min^−1^ μmol enzyme^−1^, which is dramatically more active than the previous report of 0.035 μmol min^−1^ mg^−1^ from cell lysates in *P. aeruginosa* (17). Of importance, MsuD tested here was active with C_1_, C_5_, and C_8_ alkanesulfonates (Table 1). Less activity was observed for the buffer molecules, indicating that, although each molecule could be successfully docked within ternary-MsuD, the lower enzyme activity suggests that Hepes, Pipes, and Mops bind in a less catalytically competent state.

**Table 1:** Enzyme activity of *P. fluorescens* MsuD

	Substrate					
Enzyme activity	Methane sulfonate	Octane sulfonate	Pentane sulfonate	Hepes	Pipes	Mops
Rate μmol min^−1^ μmol enzyme^−1^	26 ± 2	30 ± 3	19.4 ± 1.4	3.6 ± 1.2	4 ± 2	5 ± 2
% Relative activity	86 ± 7	100 ± 10	62 ± 5	12 ± 4	14 ± 7	19 ± 7

The desulfonation reaction was measured in a coupled reaction with 0.4 μM MsuD, 1 μM MsuE, 1 μM FMN, 500 μM NADH, and 500 μM of each respective sulfonate as detailed in Materials and Methods. The results are the average ± standard error of three determinations, and relative activity is normalized to the activity obtained for octanesulfonate.

### C terminus interaction with the identified sulfonate-binding loop

Besides length, the overall sequences of the C termini within group C flavin-dependent monooxygenases lack any apparent conservation. However, their sequences contain multiple proline, glycine, and alanine residues, which are indicative that these segments can adopt sharp bends and turns (Fig. 3 and Fig. S2). In MsuD, five prolines enable the C terminus to adopt a kinked and winding path between protein molecules. Within the C-terminal sequence are three conserved amino acids with SsuD whose side chains appear important for binding (Fig. 6*C*). Residues N372 and D373 are presented to interact with conserved side chains of R51, D110, E113, and R262. The third residue, E368, extends directly into the active site and is positioned to interact with Y330 and R296. The importance of R296 in the sulfonate-binding loop suggests that ordering of the C terminus within an adjacent protomer’s active site is linked to binding of both flavin and MS^−^.

To further test the importance of the protein C terminus, a truncation mutation of MsuD was generated in which the last 16 residues were deleted (MsuD^ΔC–16^) by means of incorporating a stop codon (Fig. S7). It is surprising that the truncated MsuD variant showed lower detected sulfite formation at 0.45 ± 0.01 μmol min^−1^ μmol enzyme^−1^ MsuD^ΔC–16^ under conditions demonstrating activity for wildtype MsuD (5.7± 0.02 μmol min^−1^ μmol enzyme^−1^). Therefore, the C terminus of MsuD has three previously unidentified roles in monooxygenase function: contributing to the tetramer interface, ordering active site residues for MS^−^ binding and catalysis, and closing off the active site from solvent in conjunction with the lid region.

## Discussion

Flavin-dependent monooxygenases catalyze a breadth of oxygenation reactions, and group C flavin-dependent monooxygenase reactions include light emission, Baeyer-Villiger oxidation, epoxidation, sulfoxidation, hydroxylation, and desulfurization (1). Despite their chemical utility, there are many outstanding questions regarding how these enzymes function at the molecular level, in part due to the limited structures for group C monooxygenases with substrates bound. The structures of MsuD reported here represent the first crystal structure of MsuD, and we have obtained crystallographic snapshots of three protein states: before flavin binding, after flavin binding, and after flavin and MS^−^ binding.

Previous studies with MsuD from *P. aeruginosa* (87% sequence identity) showed desulfonation rates of larger sulfonates between 1.3% and 31% the rate for MS^−^ (Table S5) (17), therefore MsuD was identified as a methanesulfonate monooxygenase. Our results, however, support MsuD from *P. fluorescens* as an alkanesulfonate monooxygenase with similar activity against both small (C_1_) and medium (C_5_ and C_8_) alkanesulfonates. SsuD, in comparison, shows little activity against a C1 substrate but a range of activity with medium and larger substrates (26). The active site residues within MsuD from *P. aeruginosa* and *P. fluorescens*, as well as for *E. coli* SsuD, are conserved; therefore, it is likely that residues outside of the primary active site define substrate preference.

MsuD and other group C monooxygenases adopt a (β/α)_8_ TIM barrel fold containing insertions involved in oligomerization and catalysis. In MsuD, IS-2 and IS-3 are involved in forming the MsuD homodimer, and two homodimers arrange to form a dimer-of-dimers architecture. Truncation of the C terminus of MsuD yielded a dimeric enzyme with decreased function, indicating that MsuD activity is dependent on tetramerization. Homotetramers are only observed for MsuD, SsuD (33), and BdsA (32), all of which catalyze C–S bond cleavage. DmoA similarly catalyzes C–S bond cleavage of dimethylsulfide; however, unliganded DmoA is reported to be a homodimer (30). The similar or conserved residues within group C flavin-dependent monooxygenases primarily are involved in flavin and dioxygen binding. No sequence conservation is observed in the MS^−^-binding site outside of alignment with SsuD, attesting to the diverse scope of substrates recognized by group C flavin-dependent monooxygenases.

The structures of MsuD with and without ligands support ordered binding for FMNH^−^ and MS^−^, and the preferential binding of FMN first within chains A/C and E/G is suggestive of possible cooperativity. Without ligands, the active site lid, the sulfonate-binding loop, and the protein C terminus are disordered. With FMN added, we observe binding of FMN within a subset of MsuD active sites that increases with higher concentrations of FMN. Binding of FMN leads to a partial ordering of the active site lid within some MsuD protomers, but it is not until MS^−^ (or a mimic such as succinate) is also bound that ordering of the sulfonate-binding loop, lid, and the protein C-terminal tail occurs, fully enclosing the MsuD active site, consistent with biochemical observations for the SsuD system (47, 49, 50). Studies in SsuD highlight the lid’s importance in the production of sulfite and protecting bound FMNH^−^ from oxidation (51). An analogous lid region is observed in all homologs of MsuD containing between three and five helices (Fig. S8*A*), and structures of EDTA monooxygenase EmoA (48) similarly describe an open and closed lid conformation that is linked to FMN binding. Therefore, flavin binding likely stabilizes the closed conformation of the lid in a shared general mechanism for linking flavin binding to lid closure prior to catalysis within this enzyme family (44). It remains to be seen if other group C flavin-dependent monooxygenases will utilize their extended protein C termini. In the crystal structures of MsuD homologs, the C terminus is either entirely disordered or partially built but in a different orientation (Fig. S8*B*). Truncation of the MsuD C terminus resulted in a 13-fold decrease in MS^−^ conversion to sulfite. Indeed, structures reveal that the protein C terminus donates E368 as a second coordination sphere contact to the MS^−^-binding site that is conserved in SsuD. The C terminus is highly flexible, consistent with a role in closing off the active site during catalysis. A tight interaction would interfere with substrate and product diffusion. It is possible that the observed interactions of the C terminus are a mechanism for sensing active site lid closure as a result of flavin and MS^−^ binding. Alternatively, only when the C terminus is ordered was succinate, a substrate mimic, observed in the sulfonate-binding site, implying the C terminus may play a role in sulfonate affinity. Future studies are needed to explore the role of the MsuD C terminus and the possible cooperativity in flavin binding.

**Figure 7:**
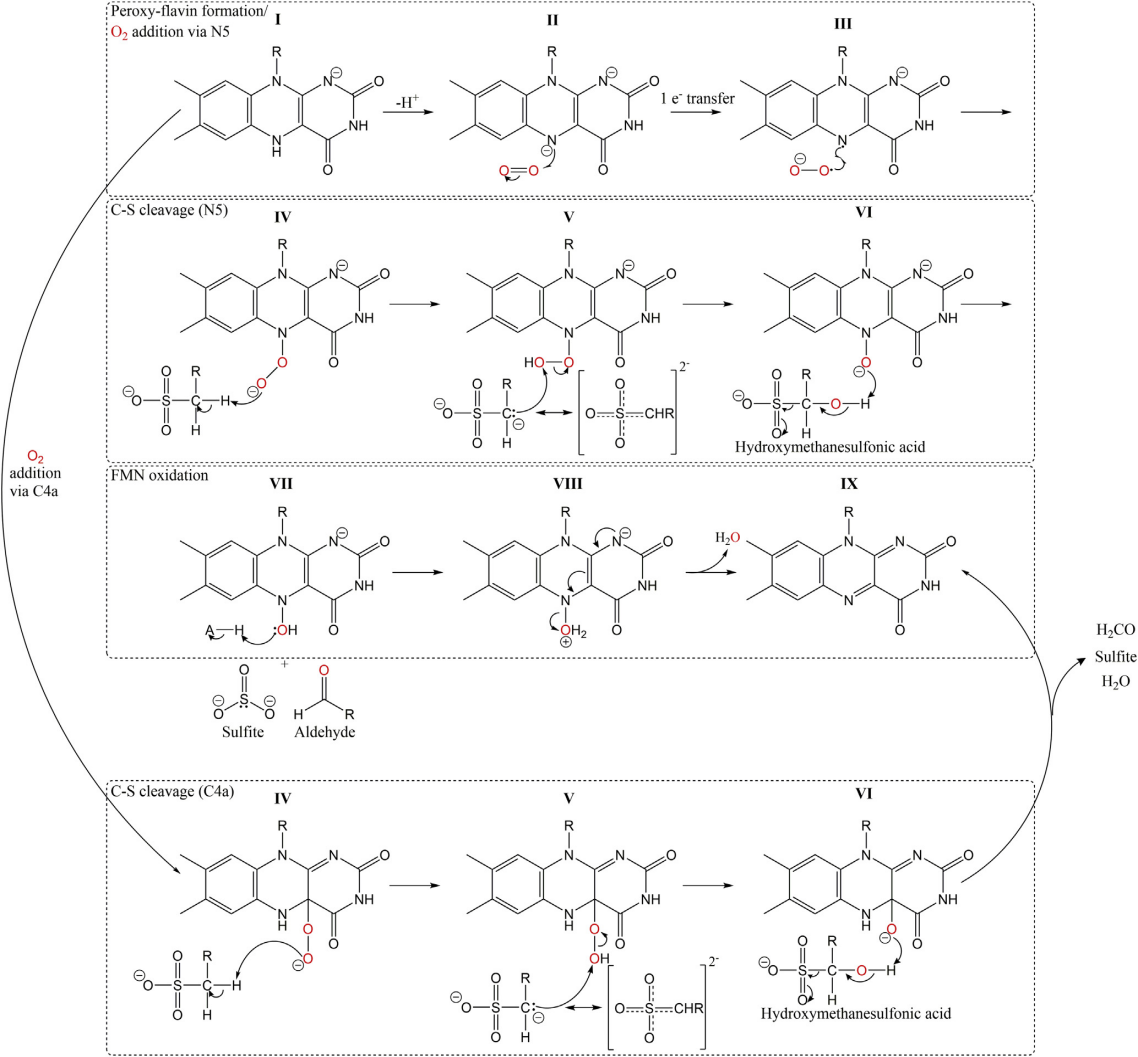
**Proposed mechanism for two-component flavin-dependent alkanesulfonate monooxygenases based on the Ternary-MsuD structure.** Dioxygen is colored *red*, and R can be a hydrogen or larger alkyl chain. The proton donor AH is likely a water molecule. Alternatively, hydroxymethanesulfonic acid may nonenzymatically convert to sulfite and aldehyde. Positioning of MS^−^ is consistent with oxygenation at the methyl, with both the N5- and C4a-pathways included for comparison. Reduced flavin is generated by the NADH:FMN oxidoreductase MsuE (not depicted).

The structure of ternary-MsuD enables us to envision how the components of an alkanesulfonate monooxygenase are oriented for catalysis and to revisit the proposed chemical mechanism. The classical monooxygenase proposed mechanism invokes formation of a C4a-(hydro)peroxyflavin intermediate (3) capable of reacting with MS^−^ (Fig. 7); however, the structure identifies the reactive dioxygen motif recently reported by Matthews *et al.* (35) on the *re*-face of the flavin. Studies of RutA support formation of an N5-peroxyflavin intermediate from dioxygen bound on the *re*-face, and inversion at N5 would bring the reactive peroxy moiety within reaction distance to the substrate bound on the *si*-face. This model demonstrates how MsuD and related monooxygenases can have variable substrate-binding sites on the *si*-face and still retain the residues needed for dioxygen binding on the opposite side of the flavin. The reactive dioxygen motif of MsuD is conserved in the positioning of residues in RutA, except for a shift of L46 and N104 closer to the N5 position of the oxidized flavin. Therefore, the dioxygen-binding site in ternary-MsuD appears to be partially occluded, which is likely a result of having the product, oxidized FMN, bound in place of the substrate, reduced FMN. Nonetheless, there are no additional pockets on the *re*-face of the flavin for dioxygen to bind, and a large active site cavity where variable alkanesulfonates would bind is present on the *si*-face without any residues to position dioxygen to react with FMNH^−^ at the C4a position; therefore, dioxygen is predicted to bind in a similar mode as observed in RutA, positioned to form an N5-peroxyflavin (Steps I to III, Fig. 7). Within the substrate alkanesulfonate there are only two possible reactive sites: the C_1_ position and the sulfur (49). As the product is sulfite, it would be unexpected for the peroxyflavin to directly attack the sulfur (52). The methyl group of MS^−^ is closest to the N5 position of FMN at 3.4 Å apart, supporting the C_1_ position as the reactive site. Therefore, the N5-peroxyflavin can deprotonate the C_1_ position, generating a resonance stabilized carbanion that can in turn attack the generated N5-hydroperoxy flavin, yielding 1-hydroxyalkanesulfonate and a reduced flavin-N5-oxide (Steps IV and V, Fig. 7). At this point the MsuD mechanism has diverged from the general proposed mechanisms for RutA, DszA, and HcbA1 (35), in which the organic substrates and products are in the same oxidation state and the cosubstrate FMNH^−^ forms flavin-N5-oxide; a reducing equivalent of NADH is needed to reform FMN (52, 53, 54). However, in MsuD the starting alkanesulfonate is more reduced than the product, formaldehyde, and the cosubstrate FMNH^−^ stays in its reduced form after hydroxylation. In effect, two more electrons must be lost from the substrate MS^−^ in comparison with the RutA, DszA, and HcbA1 reactions, and these electrons enable the formation of reduced flavin-N5-oxide, which can reform FMN after protonation and loss of water, precluding the need for an additional NADH molecule (Steps VII to IX, Fig. 7, and Fig. S9). This mechanistic scheme requires two protonation events. The generated 1-hydroxyalkanesulfonate could serve as a proton source in Step VI or alternatively convert to sulfite and an aldehyde nonenzymatically as has been proposed for the degradation of *n*-alkane-1-sulphonates (55) and taurine (9). No residues are observed in the ternary-MsuD structure to be near the C_1_-alkanesulfonate position, and N104 is the only polar residue near the N5 position of FMN. Considering asparagine is not a strong general acid, solvent is the most likely proton donor. After catalysis completes, the mobile C terminus and active site lid must move to open the active site to bulk solvent and allow product release.

Our mechanistic proposal is consistent with previous kinetic isotope experiments (50) and biochemical studies of SsuD (47). A primary kinetic isotope effect for C_1_ deuterated octanesulfonate in SsuD supports deprotonation of C_1_ as the rate-limiting step, and solvent isotope effect studies support a protonation event after the rate-limiting step. The pH profile of SsuD identified the need for a deprotonated group with a p*K*_a_ value of 6.6 and a protonated group with a p*K*_a_ value of 9.5. The ternary-MsuD structure does not reveal any residues that could serve as the source for either p*K*_a_ value, leaving only flavin as a candidate. The higher p*K*_a_ value is near the reported p*K*_a_ of 9.8 for the C4a-(hydro)peroxyflavin group of *p*-hydroxyphenylacetate-3-hydroxylase (56), suggesting that an N5-(hydro)peroxyflavin may have a similar p*K*_a_. The p*K*_a_ of free FMNH_2_ is reported to be 6.7 (57); therefore, the need for FMNH^−^ matches the lower p*K*_a_. The ribityl moiety of FMN within MsuD is in a compact state that positions the 3′-OH to form an intramolecular hydrogen bond with N1 of the isoalloxazine ring. The binding mode of FMN connects to the active site lid, the MS^−^-binding site, the protein C terminus, and the proposed site for dioxygen binding; therefore, maintenance of the ribityl moiety in a catalytically competent configuration may serve as the source of the acidic limb on the pH profile of SsuD. Currently, it is unclear why MsuD and SsuD have different preferences in substrate size, and future studies will be necessary.

The series of crystal structures of MsuD from *P. fluorescens* provides a timely addition to enzymes proposed to use an N5-(hydro)peroxyflavin intermediate, and the structure of ternary-MsuD has enabled us to revisit the alkanesulfonate monooxygenase reaction. Although we cannot fully rule out use of a C4a-(hydro)peroxyflavin intermediate, the separation of the MS^−^-binding site from the predicted oxygen-binding motif residues is consistent with a mechanism that engages both N5-peroxyflavin and N5-hydroperoxyflavin intermediates in the hydroxylation at the C_1_ position of an alkanesulfonate, and future work to explore possible intermediates in the reaction will be illuminating. Alkanesulfonates represent a family of substrates that undergo oxidation to an aldehyde and sulfite; therefore, unlike the reactions of the group C two-component flavin-dependent monooxygenases RutA, DszA, and HcbA1, a second equivalent of NADH would not be necessary to complete the catalytic cycle. Rather, oxidation of the alkanesulfonate substrate is the likely source of electrons. The structures of MsuD characterize movements of the protein linked to FMN and MS^−^ binding, including ordering of a sulfonate-binding loop and active site lid, as well as identify a novel function for the extended C terminus of the monooxygenase in closing off the enzyme active site after MS^−^ binds and maintaining the functional tetramer.

## Experimental procedures

### Materials

Recombinant DNA, molecular cloning, and microbiological procedures were carried out using standard procedures (58). PCR primers were supplied from Integrated DNA Technologies and Life Technologies Corporation, and *P. fluorescens* was kindly made available by Katharina Ribbeck from the Department of Biological Engineering at MIT. GoTaq green master mix, blue/orange 6x loading dye, 1 kb DNA ladder, and LigaFast Rapid DNA Ligation System were purchased from Promega. Alkaline phosphatase was purchased from Roche Applied Science. Chemically competent *E. coli* cells were purchased from Invitrogen. DNA purification kits were purchased from Qiagen and New England Biolabs and used as instructed. Restriction enzymes, polynucleotide kinase, and T4 DNA ligase were purchased from New England Biolabs. Ni Sepharose resin was purchased from GE Healthcare Biosciences. Expression vector pET28a was purchased from Novagen. All crystallization reagents were purchased from Hampton Research, and other chemicals were purchased from commercial suppliers and used as received.

### Construction of the MsuD_Pflu_ expression plasmid

The gene *Pfl01_3916* (GenBank accession ABA75653.1) was amplified by colony polymerase chain reaction (PCR) using GoTaq green master mix and the DNA primers shown in Table S6 for cloning into expression vector pET28a. The amplified DNA was analyzed by agarose gel electrophoresis and visualized using ethidium bromide staining. Crude PCR product was purified using a Qiagen QIAquick spin purification kit and stored at −20 °C. The amplified DNA and pET28a were digested with restriction enzymes NheI and HindIII, purified as described above, and ligated using the LigaFast Rapid DNA Ligation System. The resulting pET28msuD_Pflu_ vector was transformed into XL10 *E. coli* cells. Colonies were screened by PCR, and amplified DNA was analyzed by agarose gel electrophoresis. Colonies that exhibited amplified DNA consistent with the desired gene were used to inoculate 5 ml sterile LB broth with kanamycin sulfate (50 μg/ml) in 10 ml culture tubes and incubated overnight at 37 °C and 250 rpm. The culture tubes were centrifuged for 20 min at 3000*g* and 4 °C, and the supernatant was decanted. The plasmid DNA of the pelleted cells was isolated and purified using a Qiagen QIAprep spin miniprep kit and stored at −20 °C. The produced pET28msuD_Pflu_ vector was verified by Sanger sequencing (MIT Biopolymers Laboratory).

### Generation of MsuD_Pflu_ variants

MsuD mutants W195A, R225A, R296A and the truncation of 16 residues from the C terminus (MsuD^ΔC–16^) were constructed by site-directed mutagenesis of the previously described MsuD plasmid with designed primers listed in Table S6. PCR products were treated in reactions containing approximately 2.5 ng/μl of DNA, polynucleotide kinase, T4 DNA ligase, and T4 DNA ligase reaction buffer, and the ligated plasmids were used to transform T7 express and BL21(DE3) cells. Overnight 10 ml cultures containing LB broth with 50 μg/ml kanamycin sulfate were inoculated with single bacterial colonies and allowed to grow overnight at 37 °C and 250 rpm. Cell cultures were harvested by centrifugation for 20 min at 3000*g* and 4 °C, and the supernatant was decanted. Plasmid DNA was isolated and purified using the Monarch Plasmid Miniprep Kit, and the desired mutations were verified by Sanger sequencing (Quintara Biosciences).

### Production and purification of MsuD and MsuD variants

A starter culture (10 ml) of *E. coli* BL21(DE3) cells transformed with pET28msuD_Pflu_ was transferred into 1 l lysogeny broth (LB)-Miller containing 50 μg/ml kanamycin sulfate. The culture was grown at 37 °C with shaking at 250 rpm until the OD_600_ reached between 0.5 and 0.6, at which point the culture was cooled on ice. Cells were induced with isopropyl β-D-1-thiogalactopyranoside (IPTG) at a final concentration of 50 μM and allowed to grow overnight at 16 °C with shaking at 250 rpm prior to harvesting at 3000*g*, 4 °C for 15 min. The cell pellets were resuspended in 10 ml of wash buffer (20 mM Tris, 100 mM NaCl, pH 8.0) and centrifuged at 10,000*g*, 4 °C for 10 min. The pellets were transferred to a 50-ml Falcon tube to yield approximately 4.0 g wet cell paste, frozen in liquid N_2_, and stored at −80 °C.

The harvested cell paste was thawed, resuspended in 22 ml of lysis buffer (20 mM Tris, 500 mM NaCl, pH 8.0), and supplemented with 5 μg/ml lysozyme, 10 μg/ml RNase A, 10 μg/ml DNase I, and a Pierce protease inhibitor tablet (Thermo Fisher Scientific). The cells were lysed at 24,000 psi *via* three passages through an Avestin EmulsiFlex-B15 high-pressure homogenizer. After centrifugation at 10,000*g*, 4 °C for 20 min, the supernatant was loaded at 4 °C onto a 2.1-cm^3^ hand-packed Ni Sepharose gravity column preequilibrated with lysis buffer. The column was washed with ten column volumes of wash buffer (20 mM Tris, 300 mM NaCl, 20 mM imidazole, pH 8.0). MsuD was eluted with six column volumes of elution buffer (20 mM Tris, 500 mM NaCl, 250 mM imidazole, 10% (w/v) glycerol, pH 8.0), and fractions were qualitatively analyzed for protein using the Bradford method and sodium dodecyl sulfate polyacrylamide gel electrophoresis (SDS-PAGE). Fractions containing protein were combined and buffer exchanged into storage buffer (20 mM Tris, 100 mM NaCl, pH 8.0) and concentrated using a 30,000-Da MWCO centrifugal filter unit (MilliporeSigma). MsuD was flash frozen in liquid nitrogen and stored at −80 °C temporarily prior to purification over a HiLoad Superdex 75 pg 16/600 column (GE Scientific) equilibrated with size exclusion buffer (50 mM Tris, 100 mM NaCl, pH 8.0). Protein-containing fractions were analyzed *via* SDS-PAGE, confirming a purity >90% *via* ImageJ analysis (59). The protein fractions were combined and concentrated to 8 mg/ml with a 10-kDa centrifugal filter (Vivaspin) at 4 °C, aliquoted, flash frozen in liquid nitrogen, and stored at −80 °C. Protein concentration was determined by absorbance at 280 nm, using the calculated molar absorptivity 52,370 M^−1^ cm^−1^ (60).

The MsuD variants were expressed and purified in the same manner as wildtype MsuD, except using a Nuvia 1-ml column (Bio-Rad) for the first purification step. Protein concentrations were determined using the calculated molar absorptivity value of 46,870 M^−1^ cm^−1^ for W195A and 52,370 M^−1^ cm^−1^ for MsuD^ΔC–16^, R225A, and the R296A mutants (60). The protein fractions were combined and concentrated to between 100 and 750 μM with a 10-kDa centrifugal filter (Vivaspin) at 4 °C, aliquoted, flash frozen in liquid nitrogen, and stored at −80 °C.

### Activity assays for MsuD

MsuD variants were assayed against MS^−^ in the presence of recombinantly produced MsuE (27), and the liberation of sulfite was monitored by reaction with 5,5-dithio-bis-(2-nitrobenzoic acid) (DTNB) (17) using the molar extinction coefficient of the TNB anion of 14.1 mM^−1^ cm^−1^ (61). Reactions of 400 μM MS^−^, 1 μM MsuE, 1 μM FMN, 100 mM NaCl, and 1 μM MsuD in a buffer of 50 mM Tris-HCl at pH 7.5 were prepared in a multiwell plate with five replicates. Reactions were initiated by the addition of NADH to a final concentration of 500 μM, and final reactions contained a volume of 100 μl and were allowed to run for 5 min with shaking at 1000 rpm in a MixMate (Eppendorf) before quenching with final concentration 2 M urea. DTNB was added to each well to a final concentration of 1 mM, and absorbance at 412 nm was measured using the SpectraMax i3x Multi-Mode Microplate Reader (Molecular Devices). The background reaction of MS^−^ in the presence of MsuE, NADH, and FMN, but lacking MsuD, was subtracted to account for nonenzymatic reaction of FMNH^−^ with oxygen and MS^−^. Reactions were repeated with 10 μM FMN and 3 μM MsuE to ensure that lack of reduced FMN was not a limiting factor to the assay.

To explore the substrate profile of MsuD, MsuD was assayed against MS^−^, pentanesulfonate, octanesulfonate, Pipes, Mops, and Hepes as detailed above with minor modifications. Reactions contained 500 μM sulfonate, 500 μM NADH, 1 μM FMN, 100 mM NaCl, and 0.4 μM MsuD in a buffer of 50 mM Tris-HCl at pH 7.5 and were initiated by the addition of MsuE to a final concentration of 1 μM, and final reactions contained a volume of 100 μl and were allowed to run for 3 or 5 min with shaking at 500 rpm in a MixMate (Eppendorf) before quenching with 50 μl of 2 M urea. A longer reaction time was used for the buffer molecules, which showed lower rates of sulfite production by MsuD. The background reaction of each sulfonate in the presence of MsuE, NADH, and FMN, but lacking MsuD, was subtracted to account for nonenzymatic reaction of FMNH^−^ with oxygen and any sulfonate substrates. A control of each substrate in buffer was reacted with DTNB under the same conditions to account for any background sulfite from the commercial supplier.

### Analytical gel filtration of MsuD and MsuD^ΔC–16^

Purified MsuD and high-molecular-weight standards (Cytiva) were injected using a 100-μl loop at 4 °C onto an Enrich SEC 650 10 × 300 column (Bio-Rad). The column was preequilibrated with 50 mM sodium phosphate and 150 mM NaCl at pH 7.2. Protein sample runs were performed at 0.75 ml/min and monitored at 280 nm.

### Crystallization of MsuD

Initial crystallization conditions were identified in the polyethylene glycol (PEG)/ion HT Screen using a Phoenix pipetting robot (Art Robbins Instruments). Rectangular crystals formed within 24 to 48 h at 18 °C. Optimal crystals were obtained by sitting drop vapor diffusion in reservoir conditions of 12% to 18% (w/v) PEG 3350, and either 0.14 to 0.20 M succinate or 0.2 to 0.3 M sodium acetate set up at room temperature. Protein samples were prepared by spin filtration using a 0.22-μm Millipore CL Centrifugal Filter Unit. Drops at a ratio of 1 μl MsuD (8 mg/ml) to 1 μl reservoir were equilibrated against 600 μl of reservoir. Crystals ranging in size from 50 to 200 μm in the longest dimension were cryoprotected with increasing increments of glycerol to a final concentration of 15% to 20% (v/v) with an increase of 3% to 5% (w/v) PEG 3350. Crystals were mounted in loops and cryocooled in liquid nitrogen.

### Crystallization of the binary-soak MsuD and binary-titrate MsuD complexes with FMN

Crystals of MsuD in complex with FMN (MsuD•FMN; referred to as binary-soak MsuD) were prepared by soaking MsuD crystals grown in the succinate conditions with reservoir solution supplemented with FMN. A volume of 2 μl reservoir solution supplemented with 2 mM FMN was added to a 1-μl drop containing MsuD crystals and incubated for over 16 h at 18 °C. For the binary-titrated MsuD structure, FMN was introduced to crystals through an incremental addition of a 2 mM FMN solution using a crystallization loop until crystals turned a pale yellow. All crystals, soaked and titrated, were cryoprotected in reservoir supplemented with 2 mM FMN and increasing increments of glycerol to a final concentration of 15% to 20% (v/v) and an increase of 3% to 5% (w/v) PEG 3350. Crystals were mounted in loops and cryocooled in liquid nitrogen.

### Crystallization of the ternary-MsuD and ternary-soak MsuD complexes with FMN and methanesulfonate

Structures of the ternary complex with FMN and MS^−^ (MsuD•FMN•MS^−^) were obtained by both soaking (ternary-soak MsuD) and cocrystallization (ternary-MsuD) experiments, yielding ternary complex structures in monoclinic and hexagonal space groups, respectively. For cocrystallization, MsuD at 8 mg/ml was incubated with 2 mM FMN and 2 mM MS^−^ on ice for 15 min prior to spin filtration using a 0.22-μm Millipore CL Centrifugal Filter Unit. Crystallization was performed in a sitting drop vapor diffusion experiment with 1 μl MsuD•FMN•MS^−^ mixture and 1 μl of reservoir (0.24 M succinate and 12% (w/v) PEG 3350). Hexagonal crystals ranging in size from 50 to 200 μm in the longest dimension formed within 24 to 48 h at 18 °C. For soaking experiments, MsuD crystals were soaked in reservoir solution supplemented with FMN and MS^−^. A volume of 2 μl reservoir solution supplemented with 2 mM FMN and 2 mM MS^−^ was added to a 1-μl drop containing MsuD crystals and incubated for over 16 h at 18 °C. Crystals were cryoprotected in the same manner as binary-MsuD, but with cryoprotectant supplemented with 2 mM FMN and 2 mM MS^−^, and cryocooled in liquid nitrogen.

### X-ray diffraction data, structure determination, and refinement

X-ray diffraction data for crystals of *P. fluorescens* MsuD were collected at the Advanced Photon Source, Argonne National Laboratory, beamlines 24ID-C and 24ID-E. Datasets were collected at 100 K on either an Eiger 16M detector or a Pilatus 6M detector, using 0.2° oscillations with an exposure time of 0.2 s. Crystals of unliganded MsuD diffracted to 2.8-Å resolution and indexed to space group *P*2_1_ with eight molecules per asymmetric unit, yielding a Matthew’s coefficient of 2.43 Å^3^/Da and solvent content of 49.4%. Soaked crystals of binary-MsuD and ternary-MsuD complexes diffracted to approximately 2.8-Å resolution, with the same unit cell parameters and symmetry as unliganded MsuD crystals. Cocrystals of the ternary-MsuD complex diffracted to approximately 2.4-Å resolution and indexed to the higher symmetry space group of *P6*_1_ with four molecules per asymmetric unit, a Matthew’s coefficient of 2.37 Å^3^/Da, and a solvent content of 48.1%. Crystal parameters are reported in Table 2.

**Table 2:** Crystallography X-ray diffraction data collection and refinement statistics table

Parameters and Statistics	Monoclinic Unliganded MsuD	Monoclinic Binary-MsuD *titrated soak*	Monoclinic Binary-MsuD *soak*	Monoclinic Ternary-MsuD *soak*	Hexagonal Ternary-MsuD *cocrystal*
Data Processing					
APS Beamline	24-ID-E	24-ID-E	24-ID-C	24-ID-C	24-ID-C
Wavelength (Å)	0.9792	0.9792	0.9795	0.9795	0.9795
Resolution range (Å)	64.79–2.80 (2.87–2.80)^a^	65.03–2.80 (2.87–2.80)	83.16–2.76 (2.83–2.76)	82.21–2.75 (2.82–2.75)	46.24–2.39 (2.450–2.39)
Space group	*P* 1 2_1_ 1	*P* 1 2_1_ 1	*P* 1 2_1_ 1	*P* 1 2_1_ 1	*P* 6_1_
a, b, c (Å)	94.65 210.05 94.76	94.01 212.01 94.46	94.2 214.38 95.2	94.00 211.93 94.35	92.47 92.47 320.54
α, β, γ (°)	90 119.57 90	90 118.85 90	90 119.13 90	90 119.05 90	90 90 120
Total reflections	353,328 (26,137)	483,126 (40,854)	515,608 (52,841)	482,748 (35,316)	779,854 (72,207)
Unique reflections	75,178 (5636)	78,079 (5561)	83,520 (5925)	82,800 (5987)	60,776 (4265)
Multiplicity	4.7 (3.0)	6.2 (5.3)	6.2 (6.2)	5.8 (5.9)	12.8 (12.5)
Completeness (%)	94.90 (96.20)	97.90 (94.90)	98.10 (94.10)	(99.10) (97.20)	99.60 (94.50)
Mean I/σ(I)	12.33 (1.60)	8.54 (2.51)	10.81 (1.830)	11.7 (1.77)	16.01 (1.36)
*R*_*sym*_ (%)^b^	0.084 (0.943)	0.127 (0.613)	0.1057 (0.969)	0.083 (0.985)	0.120 (1.341)
*R*_*meas*_ (%)^c^	0.095 (1.008)	0.138 (0.707)	0.116 (1.078)	0.091 (0.987)	0.125 (1.415)
*R*_*pim*_ (%)^d^	0.042 (0.511)	0.054 (0.286)	0.046 (0.418)	0.037 (0.397)	0.0348 (0.396)
CC1/2 (%)^e^	0.999 (0.606)	0.989 (0.867)	0.997 (0.682)	0.998 (0.700)	0.999 (0.764)
Refinement					
Unique reflections	75,153	78,024	83,498	82,785	60,658
*R*_*work*_^f^	0.1803	0.1993	0.1798	0.1715	0.1836
*R*_*free*_	0.2212	0.2381	0.2240	0.2144	0.2208
Protein atoms	19,795	21,296	21,129	21,512	11,736
Ligands atoms	0	234	196	249	145
Solvent atoms	0	41	49	16	135
RMS(bonds) (Å)	0.003	0.005	0.002	0.006	0.002
RMS(angles) (°)	0.61	0.98	0.56	0.76	0.43
Average B-factor (Å^2^)					
All atoms	85.3	74.8	77.4	91.6	57.4
Protein	85.3	74.8	77.3	91.7	57.4
Ligand and ions	–	80.6	88.5	91.7	55.3
Waters	–	53.0	78.1	67.5	52.2
Ramachandran					
Favored (%)	97.73	97.16	97.05	98.84	96.81
Allowed (%)	2.27	2.84	2.87	1.16	3.19
Outliers (%)	0.00	0.00	0.07	0.00	0.00
Rotamer outliers (%)	1.78	1.93	2.62	1.29	1.33

a Highest resolution shell is shown in parentheses.

b *R*_*sym/merge*_ = Σ_hkl_Σ_i_|I_i_(hkl) − <I(hkl)>|/Σ_hkl_Σ_i_I_i_(hkl).

c *R*_*meas*_ = Σ_hkl_[N/(N − 1)]^1/2^Σ_i_|I_i_(hkl) − <I(hkl)>|/Σ_hkl_Σ_i_I_i_(hkl).

d *R*_*pim*_ = Σ_hkl_[1/(N − 1)]^1/2^Σ_i_|I_i_(hkl) − <I(hkl)>|/Σ_hkl_Σ_i_I_i_(hkl) where I_i_(hkl), <I(hkl)>, and N represent the intensity measurement, the mean intensity, and the redundancy for reflection hkl, respectively.

e *CC*∗ = [2*CC*_1/2_/(1 + *CC*_1/2_)]^1/2^ where CC_1/2_ is the correlation between two random halves of the datasets, each containing half of the measured intensities for each unique reflection, and CC∗ is an approximation of the correlation coefficient for a noise-free dataset.

f *R*_*work*_ = Σ|F_obs_(hkl) − F_calc_(hkl)|/Σ|F_obs_(hkl)|, where F_obs_(hkl) and F_calc_(hkl) are the observed and calculated structure factor amplitudes of 95% of the reflections used for refinement. *R*_*free*_ was calculated from the 5% of total reflections that were omitted from the refinement.

The datasets were processed using X-ray detector software (62), and data quality and model refinement statistics are reported in Table 2. Molecular replacement (MR) of the unliganded MsuD dataset was performed in PHASER (63) within Phenix (64) using the homolog SsuD from *E. coli* K12 (Protein Data Bank [PDB] ID 1NQK (33), seqID: 66.4%). The MR model was pruned using CHAINSAW (65), enabling PHASER to place eight protein chains within the asymmetric unit in the initial *P*2_1_ dataset. Each protein molecule was refined with rigid body refinement prior to defining NCS matrices for further refinement. The initial model was refined with simulated annealing, energy minimization, and grouped B-factor refinement using strict noncrystallographic symmetry constraints within Crystallography & NMR System (66), and iterative model building was performed in COOT (67). Noncrystallographic symmetry was relaxed to torsional restraints, and final refinement cycles with iterative model building were carried out in Phenix (64) and COOT (67). Waters were added toward the end of refinement, and final model refinements included TLS groups (Translation-Libration-Screw-rotation model) generated by Phenix.

For MR of datasets containing ternary-MsuD, the built chain A from unliganded MsuD (PDB ID 7JV3) was used as the initial search model. MR placed four protein chains in the *P6*_1_ asymmetric unit. FMN was added after rigid body refinement of the protein model. FMN was placed in 4/4 protein chains in the ternary-MsuD cocrystal. Subsequent rounds of refinement and iterative model building were carried out in COOT and Phenix (64, 67). Toward the end of refinement, waters and the MS^−^ ligand were placed and refined.

For MR of ternary-soak MsuD, eight protein chains were placed within the asymmetric unit in the *P*2_1_ dataset, using chain A of ternary-MsuD (PDB ID 7JW9) as an initial search model. FMN was placed in 7/8 protein chains, and MS^−^ was placed in 5/8 protein chains; chain G and chain H each have a chloride ion refined in the MS^−^ site. Refinement and model building proceeded as for ternary-MsuD. Occupancy values were refined for FMN and MS^−^ in the later stages of refinement, and final model refinements included single TLS groups per protein chain.

Phases for both binary-soak and binary-titrated MsuD were solved by Fourier synthesis using the completed ternary-soak MsuD model (PDB ID 7K14), and model building and refinement were performed as described for ternary-soak MsuD. In binary-soak MsuD, FMN was placed in 6/8 protein chains, whereas only the phosphate group of FMN could be placed in chains F and H. In binary-titrated MsuD, FMN was placed in 6/8 protein chains, and only the phosphate group of FMN could be placed in chains B and F. Succinate was refined in chains that contained an ordered C terminus interaction (chain E in binary-soak and chains A, C, and E in binary-titrated MsuD).

Within the series of liganded structures, placed FMN and MS^−^ molecules refined with B factors comparable with interacting protein residues. In molecules containing lower occupancy values, electron density is strongest for the flavin isoalloxazine ring and phosphate moieties, with weaker electron density for the ribityl moiety. In certain chains of the soaked structures the only density available for the FMN was about the phosphate moiety; therefore, a phosphate ion was placed instead. In ternary-soak MsuD a chloride ion refined best within two chains instead of MS^−^. At lower concentrations of FMN and in the absence of MS^−^, binary-titrated MsuD had appropriate electron density for succinate from the crystallization conditions within three chains where MS^−^ would have been bound. Table S3 summarizes the refined ligand occupancy information for each structure.

All models were verified with composite omit electron density maps calculated in Phenix (64) during refinement, and Ramachandran angles were calculated using MolProbity (68). Solved structures of MsuD showed two major regions of disorder in some of the protein chains, from around L246-D280 and A355-A381 at the end of the C terminus. The missing electron density for these regions was apparent in all chains in unliganded MsuD, and partially for the binary-soak and ternary-soak MsuD. Both regions were able to be modeled in the ternary-MsuD cocrystal. Unliganded MsuD is missing residues in all monomers (A:248–281 and 295–299, B:248–281 and 296–299, C:251–281 and 297–299, D:254–281 and 297–299, E: 249–281 and 295–299, F: 251–281 and 297–299, G: 254–281 and 297–299, H: 251–281 and 296–299) with 355 to 381 missing in all chains and Ramachandran favored, allowed and outliers at 98%, 2%, and 0%, respectively. Binary-soak MsuD shows similar missing residues in about half of its monomers (A:357–381, B:255–279 and 357–381, C:356–381, D: 255–280 and 357–381, E:356–381, F: 256–281 and 356–381, G: 254–280 and 356–381, H:263–280, 297–298, 355–360 and 374–381) with Ramachandran favored, allowed, and outliers at 97.05%, 2.87%, and 0.07%, respectively. Binary-soak titrate MsuD shows similar missing residues in about half of its monomers (A:357–381, B:248–280 and 378–381, C:356–381, D: 248–280 and 378–381, E: 249–281 and 356–381, F: 256–281 and 356–381, G: 251–280 and 356–381, H:251–280 and 378–381) with Ramachandran favored, allowed, and outliers at 97.16%, 2.84%, and 0.07%, respectively. The ternary soak shows similar missing residues in about four of eight of its monomers (A, C, D, E: 356–381, B: 278–279 and 356–381, F: 249–280 and 356–381, G: 254–261, 278–279 and 356–381, H: 250–271 and 375–381) with Ramachandran favored, allowed, and outliers at 98.84%, 1.16%, and 0%, respectively. The ternary cocrystal only shows disorder at the end of the C terminus in its four monomers (378–381 within all chains) with Ramachandran favored, allowed, and outliers at 96.88%, 3.12%, and 0%, respectively. Three *cis*-peptides are observed in the electron density: K167-P168, Y330-P331, and E349-P350. The region from approximately 250 to 280 missing in most models has higher B-factor values compared with the rest of the chain owing to the inherent flexibility of the region. Similarly, the C terminus from approximately 355 to 381 missing in most models is inherently more disordered compared with the rest of the model, but with ternary and binary complexes showing weak but clear electron density for the C terminus. Placements of ligands were verified using Polder omit maps calculated in Phenix (69). Final model validation was performed using Phenix and the RCSB validation server (www.rcsb.org). The FMN parameter file was generated using Phenix eLBOW with ideal FMN coordinates from the PDB, and ring plane parameters were generated using the Grade Web Server to enable the isoalloxazine moiety to bend slight, as is best defined by the electron density within MsuD. The MS^−^ parameter file was generated using the Grade Web Server (70) with a mol2 file of PDB ligand 03S prepared in Bioluminate 2020-3 (Schrödinger, LLC) (71, 72, 73).

### Bioinformatic analysis

A DELTA-BLAST of the *P. fluorescens* MsuD sequence was done at a cutoff of 40% to 60% identity, resulting in over 5000 sequences. An alignment was generated with ClustalOmega (74), and both ends of the alignment were trimmed and gaps removed. The residue frequency in the regions of the sulfonate-binding loop were analyzed, and images were generated with WebLogo (75). Genome alignments were performed using Mauve (76) within Geneious Prime 2020.2 created by Biomatters (https://www.geneious.com).

### Molecular docking

Rigid receptor molecular docking was performed in BioLuminate 2020-3 (Schrödinger, LLC). The ternary-MsuD tetramer and individual ligands were prepared for docking using the Protein Preparation tool (73), and docking was carried out in GLIDE (77). Ligands were prepared using LigPrep with states generated at pH 7.0, and the receptor grid was defined with a 15 × 15 × 15 Å^3^ inner box and a 35 × 35 × 35 Å^3^ outer box defined about the active site of MsuD chain A. The ligands explored include MS^−^, Pipes, Hepes, Mops, pentanesulfonate, and octanesulfonate.

Protein figures were generated using the software PyMOL (78), kinetics analyses and graphs were performed using OriginPro2020, and chemical mechanisms were constructed within ChemDraw 19.0 (PerkinElmer Informatics).

## Data availability

The datasets for atomic coordinates, structure factors, crystal structure, and diffraction data generated during this study have been deposited in the RCSB Protein Data Bank and are available at www.rcsb.org, accession code 7JV3 for MsuD, 7K64 for binary-titrated MsuD, 7JYB for binary-soak MsuD, 7K14 for ternary-soak MsuD, and 7JW9 for ternary-MsuD cocrystal. The plasmid generated in this study has been deposited to GenBank accession no. ABA75653.1. All remaining data are contained within the article.

## Supporting information

This article contains supporting information (17, 26, 30, 31, 35, 37, 42, 43, 45, 46, 48, 81, 82, 83, 84, 85, 86, 87).

## Conflict of interest

The authors declare that they have no conflicts of interest with the contents of this article.
