# Supplementary material for: Structures of the alkanesulfonate monooxygenase MsuD provide insight into C–S bond cleavage, substrate scope, and an unexpected role for the tetramer
Source: J Biol Chem. 2021 May 23;297(1):100823. doi: 10.1016/j.jbc.2021.100823 (PMC8234197; doi:10.1016/j.jbc.2021.100823)
Supplement: Supplemental Figures S1–S9 and Tables S1–S6 [file mmc1.pdf]

Supporting Information for

Structures of the alkanesulfonate monooxygenase MsuD provide insight into C–S bond cleavage, substrate scope, and an unexpected role for the tetramer

Jeremy J.M. Liew[1], Israa M. El Saudi[1], Son V. Nguyen[2,3], Denyce K. Wicht[2], and Daniel P. Dowling[1,*]

[1] Department of Chemistry, University of Massachusetts Boston, Boston MA 02125, USA

[2] Department of Chemistry and Biochemistry, Suffolk University, Boston MA 02108, USA

[3] Current address: Department of Chemistry, University of New Hampshire, Durham NH 03824, USA

Correspondence to Daniel P. Dowling: daniel.dowling@umb.edu (D.P.D.)

Supporting Information Table of Contents

1. **Supporting Figures:**

Figure S1. Related to Figure 2. The results of analytical size exclusion chromatography of MsuD

Figure S2. Related to Figure 3. The results of a sequence alignment of MsuD structural homologs with a minimum sequence identity cut-off of 19%

Figure S3. Related to Figure 3. The results of comparison of class C flavin-dependent monooxygenases that crystallize as tetramers

Figure S4. Related to Figure 4. The results of representative polder omit electron density maps for structures of MsuD demonstrating the presence of the active site lid, protein C-terminus, and the ligands FMN, MS^{-}, and succinate

Figure S5. Related to Figure 5. The results of analysis of residues and surrounding environment of (A) FMN and (B) MS^{-} in chain A of ternary-MsuD

Figure S6. Related to Figure 5. The results of (A) overlay of analogues of W195 from MsuD homologs and (B) overlay of FMN from different homolog structures demonstrating differences in ribityl tail positioning

Figure S7. Related to Figure 5. The purified protein samples for wild type MsuD and the W195A, R225A, R296A, and C-terminal truncation MsuD variants.

Figure S8. Related to Figure 6. The results of structural superimpositions of MsuD and homologs' (A) lid and (B) C-terminal regions

Figure S9. Related to Figure 7. The reactions of RutA, DszA, and HcbA1

2. **Supporting Tables:**

Table S1. Related to Figure 1. Genome and gene information for *msu* and *sfn* genes

Table S2. Related to Figures 3 and S2. Structural alignment results for MsuD from the DALI server, containing matches above or equal to 19% sequence identity

Table S3. Related to Figure 4. Summary of ligand occupancies and lid regions within MsuD crystal structures

Table S4. Related to Figure 5 and S6B. Ribityl tail torsion angles from different FMN-bound structures of the structural homologs of MsuD

Table S5. Related to Table 1. Relative activity of MsuD from *P. fluorescens* with different substrates compared to relative activity trends reported for MsuD from *P. aeruginosa* and SsuD in *E. coli*

Table S6. Related to Methods. Forward and reverse DNA primers used in PCR amplification of *msuD* of *P. fluorescens* Pf0-1 and in site-directed mutagenesis

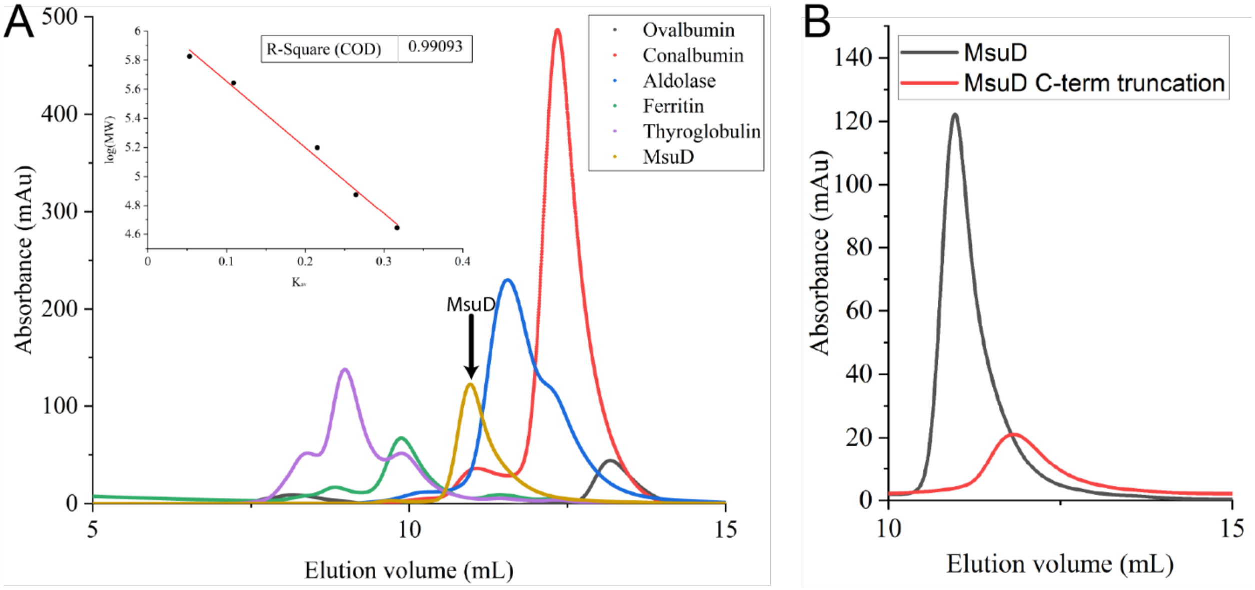

Figure S1. MsuD behaves as a tetramer in solution. (A) Size exclusion chromatography of MsuD on the ENrich SEC 650 column compared to Cytiva high-molecular weight protein standards reveals MsuD travels as an ~200 kDa species, most in line with a tetramer (monomer chain MW: 44.2 kDa, and homotetramer MW: 176.9 kDa). Molecular weight standards include: Ovalbumin (MW: 44 kDa), Conalbumin (MW: 75 kDa), Aldolase (MW: 158 kDa), Ferritin (MW: 440 kDa) and Thyroglobulin (MW: 669 kDa). (B) An overlay of wild type MsuD and the C-terminal truncation mutant (MsuD$^{\Delta C\text{-}16}$) reveal the C-terminal tail is critical for tetramerization. MsuD$^{\Delta C\text{-}16}$ behaves as an ~113 kDa species, which corresponds to a dimer.

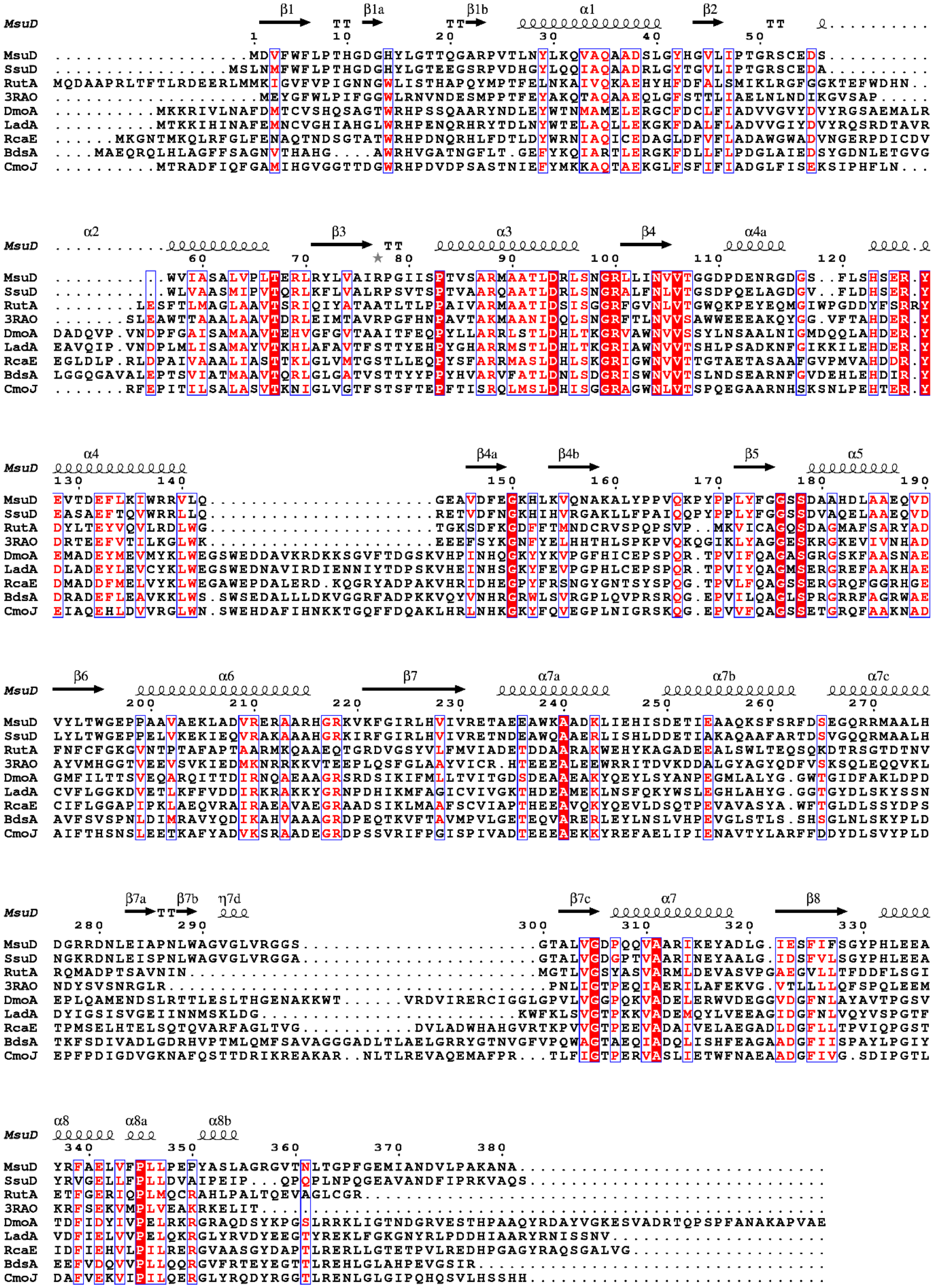
MsuD β1 β1a β1b α1 β2
1 10 20 30 40 50
MsuD ..................MDVFWFLPTHGDGHYLGTTQGARPVTLNYLKQVAQAADSLGYHGVLIPTGRSCEDS..........
SsuD ..................MSLNMFWFLPTHGDGHYLGTEEGSRPVDHGYLQQIAQAADRLGYTGVLIPTGRSCEDA..........
RutA MQDAAPRLTFTLRDEERLMMKIGVFVPIGNNGWLISTHAPQYMPTFELNKAIVQKAEHYHFDFALSMIKLRGFGGKTEFWDHN..
3RAO ....................MEYGFWLPIFGGWLRNVNDESMPPTFEYAKQTAQAAEQLGFSTTLIAELNLNDIKGVSAP.....
DmoA .........MKKRIVLNAFDMTCVSHQSAGTWRHPSSQAARYNDLEYWTNMAMELERGCFDCLFIADVVGVYDVYRGSAEMALR
LadA .........MTKKIHINAFEMNCVGHIAHGLWRHPENQRHRYTDLNYWTELAQLLEKGKFDALFLADVVGIYDVYRQSRDTAVR
RcaE .....MKGNTMKQLRFGLFENAQTNDSGTATWRHPDNQRHLFDTLDYWRNIAQICEDAGLDFVFLADAWGWADVNGERPDICDV
BdsA ....MAEQRQLHLAGFFSAGNVTHAHG....AWRHVGATNGFLT.GEFYKQIARTLERGKFDLLFLPDGLAIEDSYGDNLETGVG
CmoJ .........MTRADFIQFGAMIHGVGGTTDGWRHPDVDPSASTNIEFYMKKAQTAEKGLFSFIFIADGLFISEKSIPHFLN...
MsuD α2 β3 α3 β4 α4a
60 70 80 90 100 110 120
MsuD ...........WVIASALVPLTERLRYLVAIRPGIISPTVSARMAATLDRLSNGRLLINVVTGGDPDENRGDGS..FLSHSER.Y
SsuD ...........WLVAASMIPVTQRLKFLVALRPSVTSPTVAARQAATLDRLSNGRALFNLVTGSDPQELAGDGV..FLDHSER.Y
RutA ........LESFTLMAGLAAVTSRIQIYATAATLTLPPAIVARMAATIDSISGGRFGVNLVTGWQKPEYEQMGIWPGDDYFSRRY
3RAO .......SLEAWTTAALAAVTDRLEIMTAVRPGFHNPAVTAKMAANIDQLSNGRFTLNVVSAWWEEEAKQYGG.VFTAHDER.Y
DmoA DADQVP.VNDPFGAISAMAAVTEHVGFGVTAAITFEQPYLLARRLSTLDHLTKGRVAWNVVSSYLNSAALNIGMDQQLAHDER.Y
LadA EAVQIP.VNDPLMLISAMAYVTKHLAFAVTFSTTYEHPYGHARRMSTLDHLTKGRIAWNVVTSHLPSADKNFGIKKILEHDER.Y
RcaE EGLDLP.RLDPAIVAAALIASTTKLGLVMTGSTLLEQPYSFARRMASLDHLSKGRIGWNVVTTGTAETASAAFGVPMVAHDDR.Y
BdsA LGGQGAVALEPTSVIATMAAVTQRLGLGATVSTTYYPPYHVARVFATLDNLSDGRISWNVVTSLNDSEARNFGVDEHLEHDIR.Y
CmoJ ......RFEPITILSALASVTKNIGLVGTFSTSFTEPFTISRQLMSLDHISGGRAGWNLVTSPQEGAARNHSKSNLPEHTER.Y
MsuD α4 β4a β4b β5 α5
130 140 150 160 170 180 190
MsuD EVTDEFLKIWRRVLQ......................GEAVDFEGKHLKVQNAKALYPPVQKPYPPLYFGGSSDAAHDLAAEQVD
SsuD EASAEFTQVWRRLLQ......................RETVDFNGKHIHVRGAKLLFPAIQQPYPPLYFGGSSDVAQELAAEQVD
RutA DYLTEYVQVLRDLWG......................TGKSDFKGDFFTMNDCRVSPQPSVP..MKVICAGQSDAGMAFSARYAD
3RAO DRTEEFVTILKGLWK......................EEEFSYKGNFYELHHTHLSPKPVQKQGIKLYAGGESKRGKEVIVNHAD
DmoA EMADEYMEVMYKLWEGSWEDDAVKRDKKSGVFTDGSKVHPINHQGKYYKVPGFHICEPSPQR.TPVIFQAGASGRGSKFAASNAE
LadA DLADEYLEVCYKLWEGSWEDNAVIRDIENNIYTDPSKVHEINHSGKYFEVPGPHLCEPSPQR.TPVIYQAGMSERGREFAAKHAE
RcaE DMADDFMELVYKLWEGAWEPDALERD.KQGRYADPAKVHRIDHEGPYFRSNGYGNTSYSPQG.TPVLFQAGSSERGRQFGGRHGE
BdsA DRADEFLEAVKKLWS.SWSEDALLLDKVGGRFADPKKVQYVNHRGRWLSVRGPLQVPRSRQG.EPVILQAGLSPRGRRFAGRWAE
CmoJ EIAQEHLDVVRGLWN.SWEHDAFIHNKKTGQFFDQAKLHRLNHKGKYFQVEGPLNIGRSKQG.EPVVFQAGSSETGRQFAAKNAD
MsuD β6 α6 β7 α7a α7b α7c
200 210 220 230 240 250 260 270
MsuD VYLTWGEPPAAVAEKLADVRERAARHGRKVKFGIRLHVIVRETAEEAWKAADKLIEHISDETIEAAQKSFSRFDSEGQRRMAALH
SsuD LYLTWGEPPELVKEKIEQVRAKAAAHGRKIRFGIRLHVIVRETNDEAWQAAERLISHLDDETIAKAQAAFARTDSVGQQRMAALH
RutA FNFCFGKGVNTPTAFAPTAARMKQAAEQTGRDVGSYVLFMVIADETDDAARAKWEHYKAGADEEALSWLTEQSQKDTRSGTDTNV
3RAO AYVMHGGTVEEVSVKIEDMKNRRKKVTEEPLQSFGLAAYVICR.HTEEEALEEWRRITDVKDDALGYAGYQDFVSKSQLEQQVKL
DmoA GMFILTTSVEQARQITTDIRNQAEAAGRSRDSIKIFMLLTVITGDSDEAAEAKYQEYLSYANPEGMLALYG.GWTGIDFAKLDPD
LadA CVFLGGKDVETLKFFVDDIRKRAKKYGRNPDHIKMFAGICVIVGKTHDEAMEKLNSFQKYWSLEGHLAHYG.GGTGYDLSKYSSN
RcaE CIFLGGAPIPKLAEQVRAIRAEAVAEGRAADSIKLMAAFSCVIAPTHEEAVQKYQEVLDSQTPEVAVASYA.WFTGLDLSSYDPS
BdsA AVFSVSPNLDIMRAVYQDIKAHVAAAGRDPEQTKVFTAVMPVLGETEQVARERLEYLNSLVHPEVGLSTLS.SHSGLNLSKYPLD
CmoJ AIFTHSNSLEETKAFYADVKSRAADEGRDPSSVRIFPGISPIVADTEEEAEKKYREFAELIPIENAVTYLARFFDDYDLSVYPLD
MsuD β7a β7b η7d β7c α7 β8
280 290 300 310 320 330
MsuD DGRRDNLEIAPNLWAGVGLVRGGS.....................GTALVGDPQQVAARIKEYADLG.IESFIFSGYPHLEEA
SsuD NGKRDNLEISPNLWAGVGLVRGGA.....................GTALVGDGPTVAARINEYAALG.IDSFVLSGYPHLEEA
RutA RQMADPTSAVNIN..............................MGTLVGSYASVARMLDEVASVPGAEGVLLTFDDFLSGI
3RAO NDYSVSNRGLR................................PNLIGTPEQIAERILAFEKVG.VTLLLLQFSPQLEEM
DmoA EPLQAMENDSLRTTLESLTHGENAKKWT......VRDVIRERCIGGLGPVLVGGPQKVADELERWVDEGGVDGFNLAYAVTPGSV
LadA DYIGSISVGEIINNMSKLDG.......................KWFKLSVGTPKKVADEMQYLVEEAGIDGFNLVQYVSPGTF
RcaE TPMSELHTELSQTQVARFAGLTVG...........DVLADWHAHGVRTKPVVGTPEEVADAIVELAEGADLDGFLLTPVIQPGST
BdsA TKFSDIVADLGDRHVPTMLQMFSAVAGGGADLTLAELGRRYGTNVGFVPQWAGTAEQIADQLISHFEAGAADGFIISPAYLPGIY
CmoJ EPFPDIGDVGKNAFQSTTDRIKREAKAR..NLTLREVAQEMAFPR...TLFIGTPERVASLIETWFNAEAADGFIVG.SDIPGTL
MsuD α8 α8a α8b
340 350 360 370 380
MsuD YRFAELVFPLLPEPYASLAGRGVTNLTGPFGEMIANDVLPAKANA..............................
SsuD YRVGELLFPLLDVAIPEIP...QPQPLNPQGEAVANDFIPRKVAQS.............................
RutA ETFGERIQPLMQCRAHLPALTQEVAGLCGR...
3RAO KRFSEKVMPLVEAKRKELIT...
DmoA TDFIDYIVPELRKRGRAQDSYKPGSLRRKLIGTNDGRVESTHPAAQYRDAYVGKESVADRTQPSPFANAKAPVAE
LadA VDFIELVVPELQKRGLYRVDYEEGTYREKLFGKGNYRLPDDHIAARYRNISSNV.....................
RcaE IDFIEHVLPILRERGVAASGYDAPTLRERLLGTETPVLREDHPGAGYRAQSGALVG...................
BdsA EEFVDQVVPLLQQRGVFRTEYEGTTLREHLGLAHPEVGSIR..................................
CmoJ DAFVEKVIPILQERGLYRQDYRGGTLRENLGLGIPQHQSVLHSSHH.............................

Figure S2. Sequence alignment of MsuD structural homologs. Homologous structures identified using the DALI server with a minimum sequence identity cut-off of 19% are displayed. The protein secondary structural elements from the MsuD structure are indicated above the MsuD sequence. Blue boxes indicate conserved and similar residues, with strictly conserved residues highlighted in red and similar residues in red text. The alignment figure was prepared using the Easy Sequencing in PostScript (ESPript) online program v3.0 (1). MsuD (GenID:ABA75653.1) was aligned with SsuD (GenID: CAB40391.1, seqID: 67%), RutA (GenID:AAC74097.1, seqID: 25%), PDBID: 3RAO (GenID:AAS39998.1, seqID: 32%), DmoA (GenID:ADU77278.1, seqID: 23%), LadA (GenID:ABO68832.1, seqID: 25%), RcaE (UniProt ID: A0A3B6UEK8, seqID: 23%), BdsA (GenID:BAC20180.1, seqID: 22%), and CmoJ (GenID:CAB14891.1, seqID: 24%).

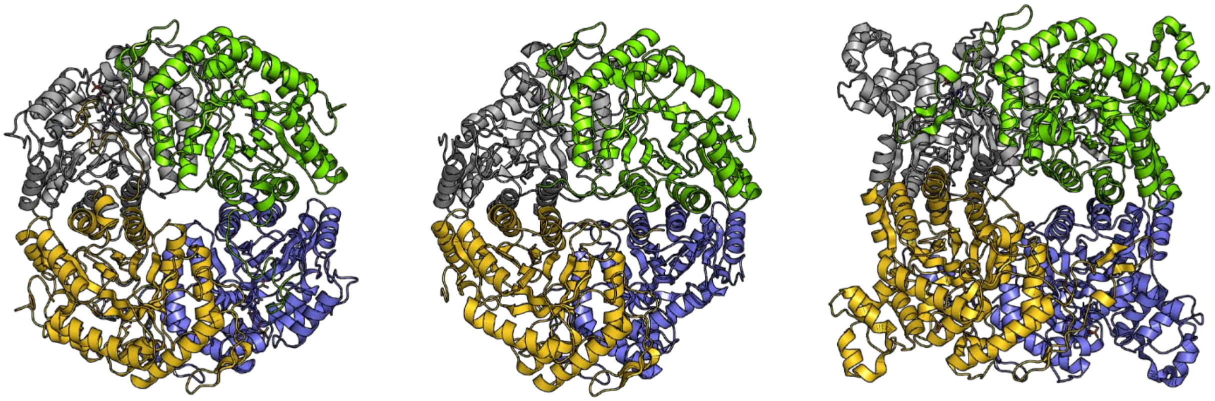

Figure S3. Comparison of class C flavin-dependent monooxygenases that crystallize as tetramers. Tetramers are displayed from left to right are MsuD (PDB ID 7JW9), SsuD (PDB ID 1NQK), and BdsA (PDB ID 5XKD). Protein chains are colored green, blue, yellow, and gray, respectively.

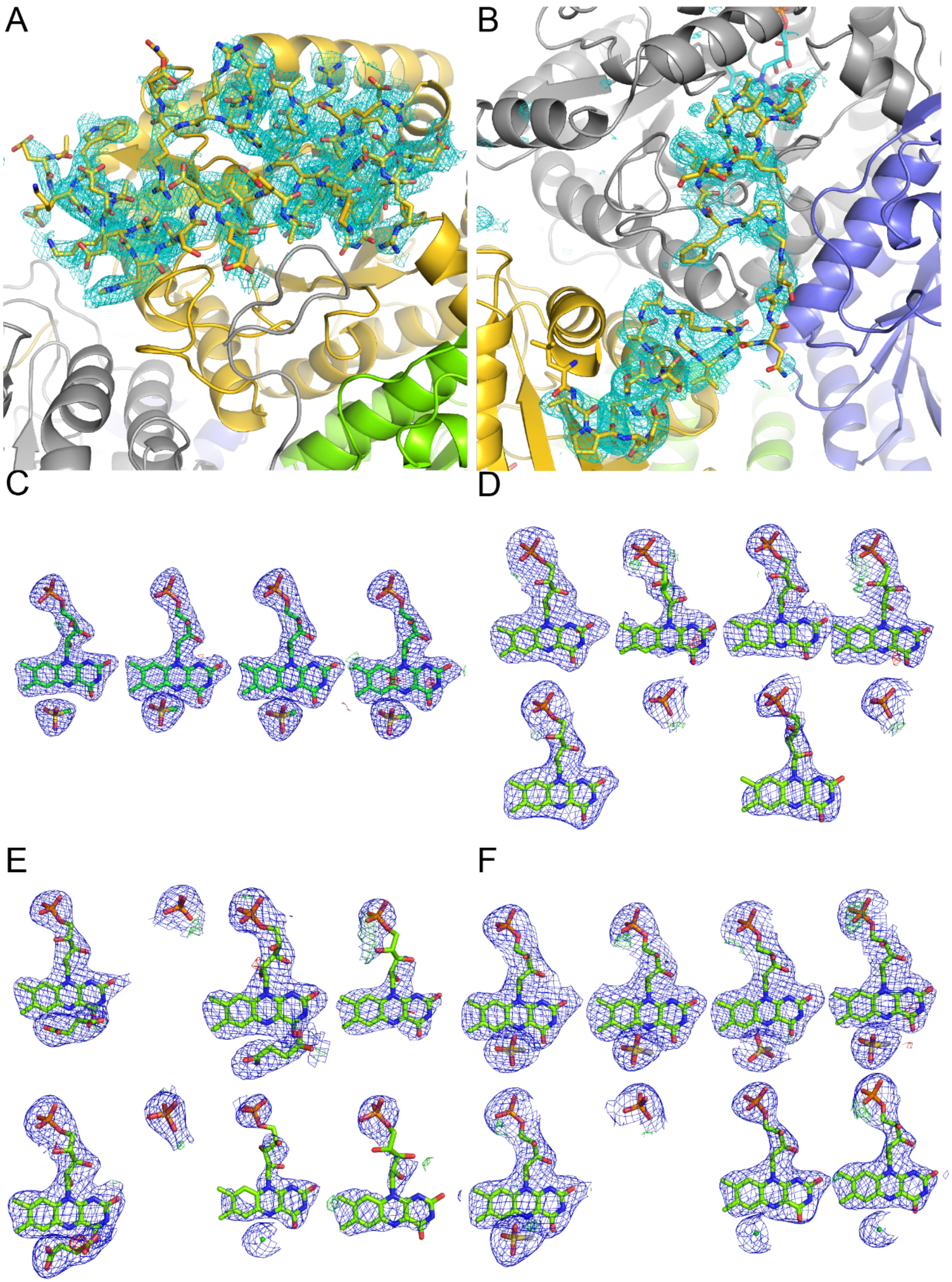

Figure S4. Representative polder omit electron density maps for structures of MsuD demonstrating the presence of the active site lid, protein C-terminus, and the ligands FMN, MS^{-},

and succinate. (A) and (B) Polder omit electron density for the best ordered lid region and C-terminus (chain A) from ternary-MsuD (cocrystal) are displayed. Protein chains A-D are colored green, blue, yellow, and gray, respectively. Polder mFo-DFc omit maps were calculated for segments of five residues at a time and are contoured at 3σ level, carved to 2 Å about the lids. Polder omit maps for ligands (FMN, MS^{-}, and succinate) are shown from chains A/B/C/D on top and E/F/G/H on the bottom of the MsuD tetramer in (C) ternary-MsuD (cocrystal), (D) binary-soak MsuD, (E) binary-titrated MsuD, and (F) ternary-soak MsuD. Omit maps for ligands are at 5σ level and Fo-Fc electron density maps at ± 3σ level, colored green for positive peaks and red for negative peaks, carved up to 2 Å about the ligands**.**

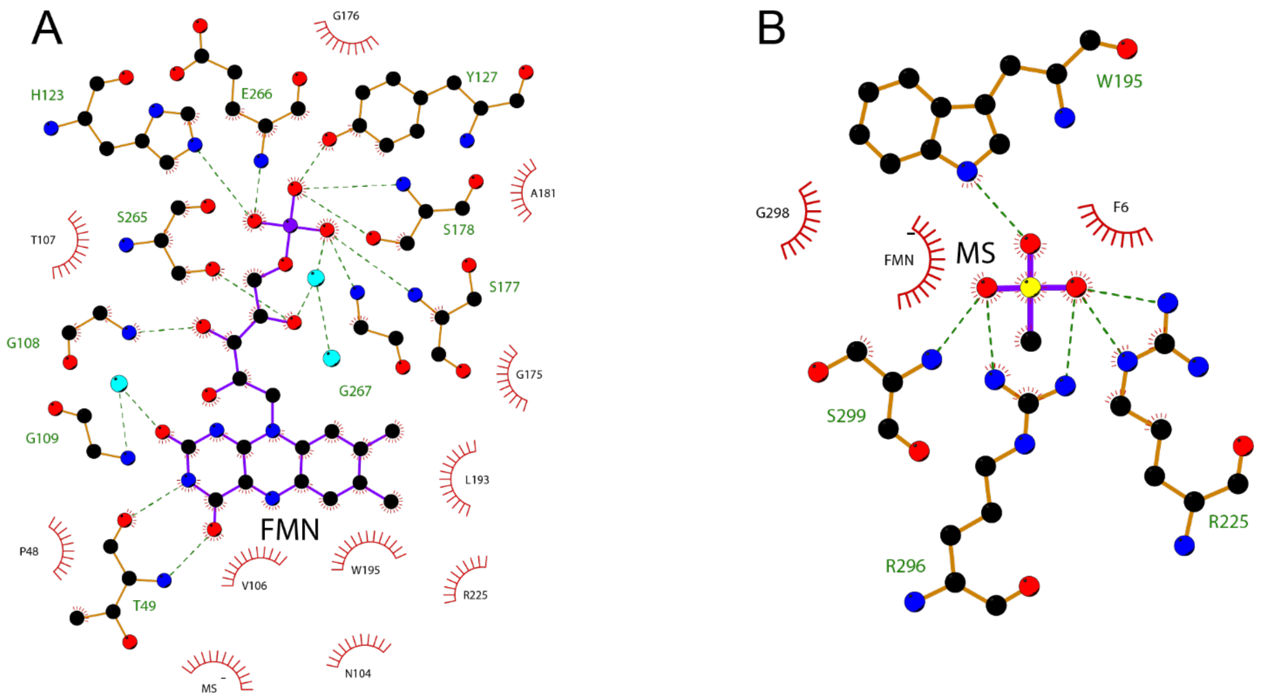

Figure S5. Analysis of residues and surrounding environment of (A) FMN and (B) MS^{-} in chain A of ternary-MsuD, showing interactions within hydrogen bonding distance and residues within van der Waals contact distances. Figure generated using LigPlot+ (2).

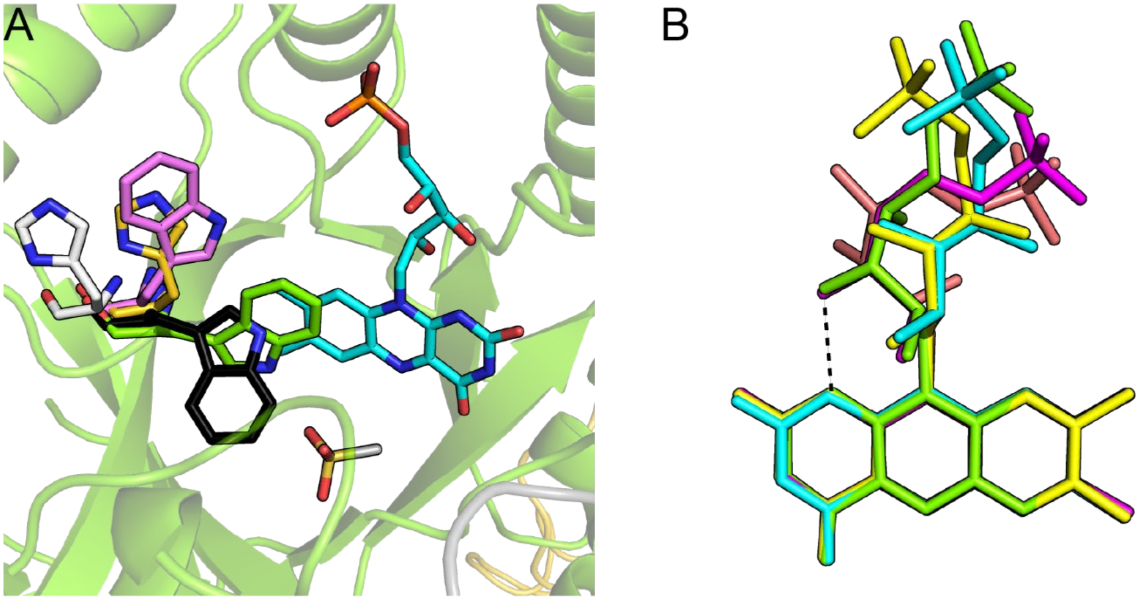

Figure S6. (A) Overlay of unliganded MsuD (black), ternary-MsuD (green), SsuD (pink), CmoJ (white), and PDBID: 3RAO (orange), demonstrating the position of aromatic residues analogous to W195 in the absence of FMN. FMN is displayed with cyan carbons from the ternary-MsuD cocrystal structure. (B) Overlay of FMN from different homolog structures demonstrating differences in ribityl tail positioning. The isoalloxazine rings of FMN were superimposed for MsuD (green, PDB ID 7JW9) with RutA (cyan, PDB ID 5WAN), LadA (purple, PDB ID 3B9O), RcaE (yellow, PDB ID 5W4Y) and BdsA (pink, PDB ID 5XKD).

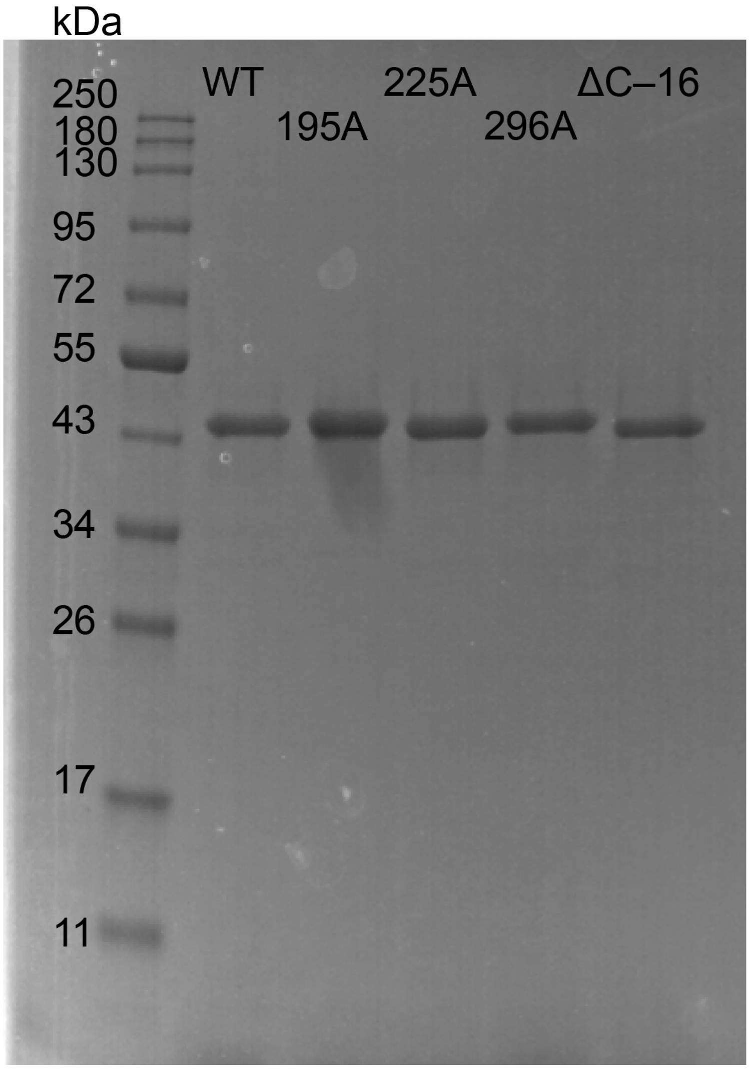

Figure S7. Recombinantly expressed MsuD variants were purified and analyzed by SDS-PAGE. Approximately 1 µg samples of fully purified MsuD including WT and variants were loaded onto a BioRad AnyKD Protein Gel and stained with SimplyBlue SafeStain. The MW sizes of the NEB Protein Ladder are labeled.

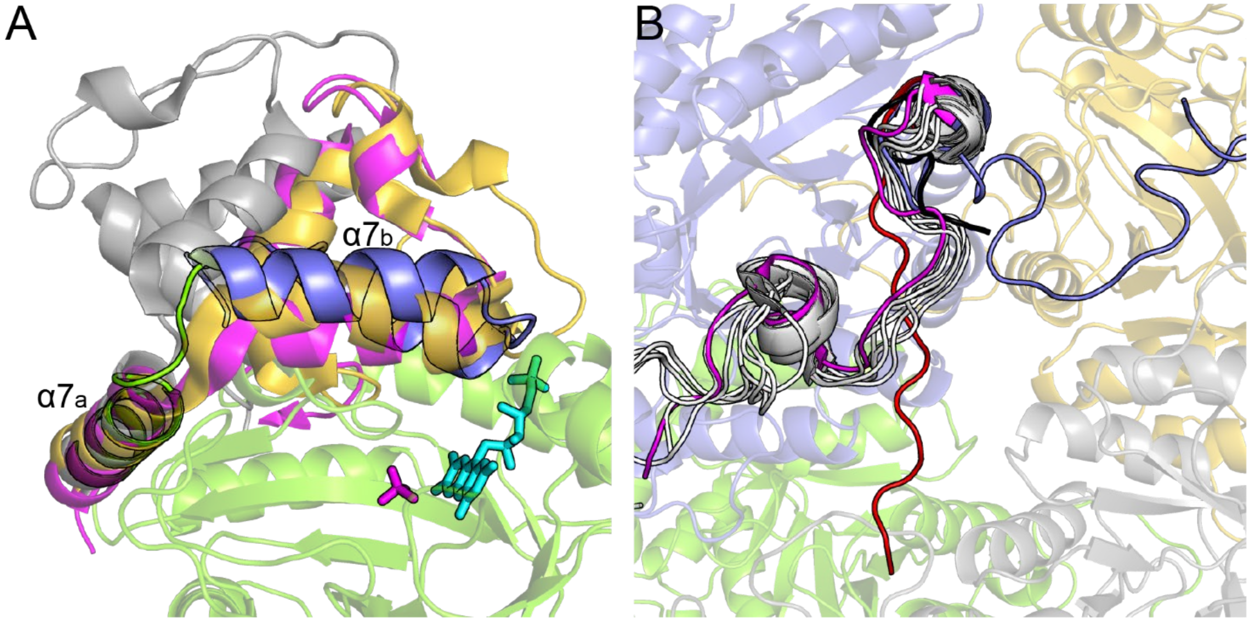

Figure S8. Structural superimpositions of MsuD homologs reveal similarities and differences in their lids and C-termini. (A) An overlay of several lids from MsuD homologs is displayed. Protein overlays are displayed as ribbons. MsuD is colored green with FMN and MS^- in cyan and magenta sticks, respectively. A variable number of helices are present in the lid region of different homologs, and an overlay demonstrates how the first helix is consistently placed. The homologs shown are RcaE (orange), LadA (magenta) and CmoJ (gray). (B) A comparison of the C-termini in MsuD and its homologs is displayed. The C-termini of the three tetrameric monooxygenase structures are displayed for MsuD (blue), SsuD (red), and BdsA (magenta), and the dimeric homologs (DmoA, EmoA, LadA, RcaE, CmoJ, and PDB ID 3SDO) are shown in white. The C-terminus in MsuD is in a conformation not previously observed.

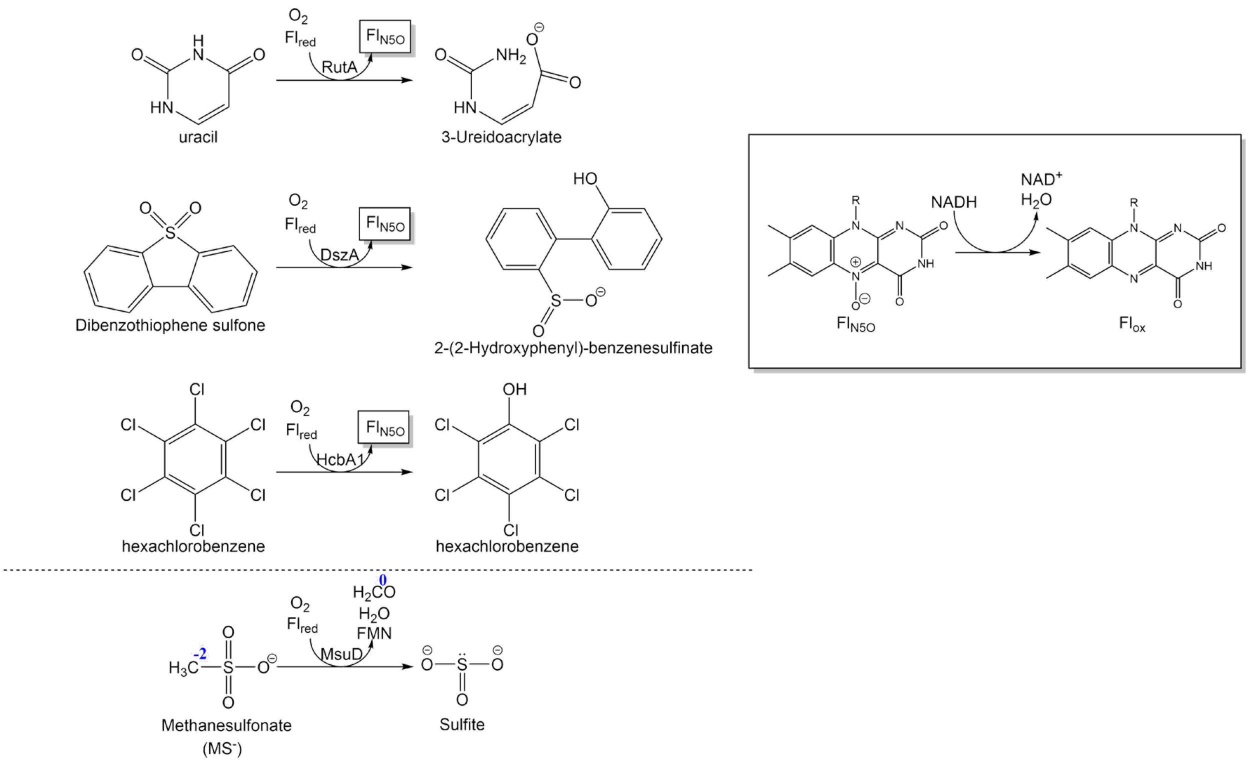

Figure S9. The proposed reactions of RutA, DszA, and HcbA1 demonstrate differences in substrate oxidation state with MsuD. Recent work by Matthew's et al have proposed the use of an N5-(hydro)peroxy (Fl_{N5OO})intermediate in the reactions of three class C two-component flavin-dependent monooxygenases, RutA, DszA, and HcbA1, and a flavin N5-oxide is formed in the reaction cycle (3). Considering that the starting substrates of RutA, DszA, and HcbA1 are uracil, dibenzothiophene sulfone, and hexachlorobenzene, respectively, these substrates are in a more oxidized state at the carbon center proposed for attack by the Fl_{N5OO} than an alkanesulfonate substrate for MsuD. Therefore, the mechanisms of RutA, DszA, and HcbA1 would generate Fl_{N5O} and must invoke an NADH molecule for the final water reduction step to regenerate oxidized flavin. Oxidation states for carbon in the MsuD reaction are shown.

Supporting Tables.

Table S1. Genome and gene information for *msu* and *sfn* genes from *P. fluorescens* P0-1, *P. aeruginosa* PAO1, and *P. putida* KT2440.

Sequence Name	Protein Name	Length	locus_tag	old_locus_tag	gene product annotation
P. aeruginosa PAO1 NC_002516	SfnR1	1131	PA2354		transcriptional regulator
	MsuC	1185	PA2355		$FMNH_2$-dependent monooxygenase
	MsuD	1146	PA2356		methanesulfonate monooxygenase
	MsuE	561	PA2357		FMN reductase MsuE
	SfnR2	1086	PA2359		transcriptional regulator
	SfnG	1095	PA3954		hypothetical protein
P. fluorescens Pf0-1 NC_007492	MsuE	564	PFL01_RS19665	Pfl01_3915	FMN reductase
	MsuD	1146	PFL01_RS19670	Pfl01_3916	$FMNH_2$-dependent alkanesulfonate monooxygenase
	MsuC	1188	PFL01_RS19675	Pfl01_3917	acyl-CoA dehydrogenase family protein
	SfnR	1104	PFL01_RS19680	Pfl01_3918	sigma-54-dependent Fis family transcriptional regulator
	SfnG	1095	PFL01_RS14520	Pfl01_2879	dimethyl sulfone monooxygenase SfnG
P. putida NC_002947	SfnR	1104	PP_RS14410	PP_2771	sigma-54-dependent Fis family transcriptional regulator
	MsuC	1188	PP_RS14415	PP_2772	acyl-CoA dehydrogenase family protein
	hypothetical protein CDS	159	PP_RS14420	PP_2773	hypothetical protein
	SfnB	1233	PP_RS14335	PP_2755	SfnB family sulfur acquisition oxidoreductase
	hypothetical protein CDS	387	PP_RS14340	PP_2756	hypothetical protein
	sugar ABC transporter substrate-binding protein CDS	972	PP_RS14345	PP_2757	sugar ABC transporter substrate-binding protein
	sugar ABC transporter substrate-binding protein CDS	1029	PP_RS14350	PP_2758	sugar ABC transporter substrate-binding protein
	sugar ABC transporter ATP-binding protein CDS	1539	PP_RS14355	PP_2759	sugar ABC transporter ATP-binding protein
	ABC transporter permease CDS	978	PP_RS14360	PP_2760	ABC transporter permease
	ABC transporter permease CDS	987	PP_RS14365	PP_2761	ABC transporter permease
	SfnA	1239	PP_RS14370	PP_2762	acyl-CoA dehydrogenase family protein
	SfnF	561	PP_RS14375	PP_2764	FMN reductase
	SfnG	1092	PP_RS14380	PP_2765	dimethyl sulfone monooxygenase SfnG

Table S2. Structural alignment results for MsuD from the DALI server (4), containing matches above or equal to 19% sequence identity. With the exceptions of RcaE, 1LUC, and 5LXE, these proteins are predicted to be class C flavin-dependent monooxygenases.

Chain	RMSD	% id	Number of aligned Cα	Name	Description	References
1NQK	1.3	69	328	SsuD	Alkanesulfonate monooxygenase	(5)
3RAO	2.0	32	320	N/A	Luciferase-like monooxygenase	(6)
5WAN	2.1	25	312	RutA	Pyrimidine monooxygenase	(7)
5W4Z	2.1	23	312	RcaE	Riboflavin lyase	(8)
3B9O	2.5	25	328	LadA	Long-chain alkane monooxygenase	(9)
5XKD	2.6	22	336	BdsA	Dibenzothiophene monooxygenase	(10)
3SDO	2.3	21	248	N/A	Nitrilotriacetate monooxyge nase	(11)
6ASL	2.7	24	248	CmoJ	Cysteine salvage pathway	(8)
5DQP	2.8	19	320	EmoA	Ethylenediaminetetraacetic acid monooxygenase	(12)
6AK1	2.8	23	328	DmoA	Dimethylsulfide monooxygenase	(13)
1LUC	2.7	19	296	N/A	Bacterial luciferase	(14)
3FGC	2.7	18	304	N/A	Bacterial luciferase	(15)
5LXE	2.4	21	288	Rh-fgd1	Glucose-6-phosphate dehydrogenase	(16)

Table S3. Summary of ligand occupancies and lid regions within MsuD crystal structures. The active site lid consists of residues D250–L282. In structures with an ordered C-terminus, the final four residues are unable to be built. For soaking experiments, FMN binding appears to be strongest in chains A/C and E/G of the two MsuD tetramers, whereas FMN binds strongly in all molecules of the cocrystal structure (highlighted yellow).

		Ternary cocrystal				Unliganded				Binary-soak				Ternary-soak				Binary-titrated			
	**Chain**	FMN	MS^-	Lid	C-terminus	FMN	MS^-	Lid	C-terminus	FMN	MS^-	Lid	C-terminus	FMN	MS^-	Lid	C-terminus	FMN	MS^-	Lid	C-terminus
Tetramer One	A	F [a]	F	O [b]	O	–	–	D	D	F	–	O	D	NF	P	O	D	NF	– [f]	O	D
	B	F	F	O	O	–	–	D	D	P	–	D	D	P	P	O	D	– [c]	–	D	O
	C	F	F	O	O	–	–	D	D	F	–	O	D	NF	P	O	D	NF	– [f]	O	D
	D	F	F	O	O	–	–	D	D	NF	–	D	D	P	P	O [g]	D	P	–	D	O
Tetramer Two	E					–	–	D	D	F	– [f]	O	D	NF	P	O	D	NF	– [f]	O	D
	F					–	–	D	D	– [c]	–	D	D	– [c]	–	D	D	– [c]	–	D	D
	G					–	–	D	D	P	–	D	D	P	– [e]	D [h]	D	P	–	D	D
	H					–	–	D	D	– [c]	–	O [d]	O	P	– [e]	D	O	P	–	D	O

[a] Due to the moderate resolution of liganded structures, occupancies of ligand are reported as fully bound (F, 100%), nearly fully bound (NF, 90% - 99%), or partially bound (P, <90%).
[b] O= ordered; D= disordered
[c] Phosphate has been placed in these chains as a substitute for the phosphate head of FMN as the rest of the FMN density is not present
[d] The lid of this chain was only partially built up to R262, denoting a potential alternate conformation
[e] A chloride ion was placed in these chains of the ternary soak
[f] Succinate was placed in these chains of the binary structures; no MS^- was used in the experiment
[g] lacks electron density for R278 and R279
[h] contains electron density for $a7_c$ only

Table S4. The ribityl tail from different FMN-bound structures of the structural homologs of MsuD demonstrate altered torsion angles. The torsion angles of the ribityl tail range from nearly all 180° in the fully extended state of CmoJ to a more compact state within BdsA.

		Torsion angle of ribityl tail (°)			
Structure	PDB ID	N10-C1'-C2'-C3'	C1'-C2'-C3'-C4'	C2'-C3'-C4'-C5'	C3'-C4'-C5'-O5'
MsuD	7JW9	-131.8	-77.4	70.7	-161.1
RutA	5WAN	178.6	-53.1	-179.9	60.8
BdsA	5XKD	124.4	67.7	42.5	-84.0
RcaE	5W4Y	-174.8	-58.3	-178.5	56.6
LadA	3B9O	-125.7	-84.2	4.7	-91.0
CmoJ	6ASL	-179.1	179.6	-177.8	-171.8
LuxA	3FGC	169.0	-74.3	-99.3	-31.4

Table S5. Comparison of activity of alkanesulfonate monooxygenases from different organisms and genes with different sulfonate group containing substrates.

			Substrates					
			Methanesulfonate	Octanesulfonate	Pentanesulfonate	HEPES	PIPES	MOPS
Organism and Gene	MsuD *P. fluorescens*	% relative activity	86 ± 7	100 ± 10	62 ± 5	12 ± 4	14 ± 7	19 ± 7
	MsuD *P. aeruginosa*[a]	% relative activity	100	N/A	13.0	9.0	31.0	17.2
	SsuD *E. coli*[b]	% relative activity[c]	1.5	100	87.3	22.9	63.1	78.6

[a] (17)
[b] (18)
[c] Octanesulfonate activity was scaled from 46.3% of 100% (4.1 µmol min^{-1} mg^{-1}) 1,3-Dioxo-2-isoindolineethanesulfonic acid activity for comparison of substrates tested in this work. Other substrates were similarly scaled.

Table S6. The *msuD* gene of *P. fluorescens* Pf0-1 was amplified by PCR using the forward and reverse DNA primers shown. The site for restriction enzyme NheI is underlined in the forward primers and the site for restriction enzyme HindIII is underlined in the reverse primers. Mutagenesis was done with forward and reverse designed primers.

**Name**	**Sequence**
Pfl01_msuD_fw (Pfl01_3916_fw)	5'- GAT ATA GCT AGC ATG GAT GTT TTC TGG TTC CTG CCG ACT CAC GGC GAC GG -3'
Pfl01_msuD_rv (Pfl01_3916_rv)	5'- GAT ATA AAG CTT TCA GGC GTT GGC TTT GGC GGG CAG TAC ATC GTT GGC G -3'
MsuD-W195A-F	5'- C GTG TAC CTG ACG GCA GGC GAA CCA CCG GC -3'
MsuD-W195A-R	5'- TCG ACC TGC TCG GCG GCC -3'
MsuD-R225A-F	5'- G TTC GGC ATC GCG CTG CAC GTG ATC GTG -3'
MsuD-R225A-R	5'- TTC ACT TTG CGC CCG TGA -3'
MsuD-R296A-F	5'- C GGT CTG GTG GCA GGC GGT TCC GGC ACT G -3'
MsuD-R296A-R	5'- ACG CCG GCC CAC AGG TTG -3'
MsuD-Stop-F	5'- TC ACC GGG CCA TGA GAC GAA ATG ATC GCC AAC G -3'
MsuD-Stop-R	5'- GGT TGG TGA CGC CGC GTC -3'

References

1. Robert, X., and Gouet, P. (2014) Deciphering key features in protein structures with the new ENDscript server. *Nucleic Acids Res.* **42**, W320-W324

2. Laskowski, R. A., and Swindells, M. B. (2011) LigPlot+: multiple ligand-protein interaction diagrams for drug discovery. *J. Chem. Inf. Model.* **51**, 2778-2786

3. Matthews, A., Saleem-Batcha, R., Sanders, J. N., Stull, F., Houk, K. N., and Teufel, R. (2020) Aminoperoxide adducts expand the catalytic repertoire of flavin monooxygenases. *Nat. Chem. Biol.* **16**, 556-563

4. Holm, L. (2020) DALI and the persistence of protein shape. *Protein Sci.* **29**, 128-140

5. Zhang, R., Skarina, T., Savchenko, A., Edwards, A., Joachimiak, A., and Midwest Center for Structural Genomics (MCSG). (2003) Structural genomics, crystal structure of alkanesulfonate monooxygenase.

6. Domagalski, M. J., Chruszcz, M., Xu, X., Cui, H., Chin, S., Savchenko, A., Edwards, A., Joachimiak, A., Minor, W., and Midwest Center for Structural Genomics (MCSG). (2011) Crystal structure of the luciferase-like monooxygenase from Bacillus cereus ATCC 10987. Protein Data Bank. RCSB, doi: http://doi.org/10.2210/pdb3RAO/pdb

7. Mukherjee, T., Zhang, Y., Abdelwahed, S., Ealick, S. E., and Begley, T. P. (2010) Catalysis of a flavoenzyme-mediated amide hydrolysis. *J. Am. Chem. Soc.* **132**, 5550-5551

8. Bhandari, D. (2017) *Studies on the flavin-mediated cysteine salvage pathway, structural enzymology of riboflavin lyase and mechanistic investigation of the radical S-adenosyl-L-methionine-mediated tryptophan lyase.* Ph.D. PhD Thesis, Texas A&M University: College Station, Texas

9. Li, L., Liu, X., Yang, W., Xu, F., Wang, W., Feng, L., Bartlam, M., Wang, L., and Rao, Z. (2008) Crystal structure of long-chain alkane monooxygenase (LadA) in complex with coenzyme FMN: unveiling the long-chain alkane hydroxylase. *J. Mol. Biol.* **376**, 453-465

10. Su, T., Su, J., Liu, S., Zhang, C., He, J., Huang, Y., Xu, S., and Gu, L. (2018) Structural and Biochemical Characterization of BdsA from Bacillus subtilis WU-S2B, a Key Enzyme in the "4S" Desulfurization Pathway. *Front. Microbiol.* **9**, 231

11. Clifton, M. C., Edwards, T. E., and Seattle Structural Genomics Center for Infectious Disease (SSGCID). (2011) Structure of a nitrilotriacetate monooxygenase from Burkholderia pseudomallei. Protein Data Bank. RCSB, doi: 10.2210/pdb3SDO/pdb

12. Jun, S. Y., Lewis, K. M., Youn, B., Xun, L., and Kang, C. (2016) Structural and biochemical characterization of EDTA monooxygenase and its physical interaction with a partner flavin reductase. *Mol. Microbiol.* **100**, 989-1003

13. Cao, H. Y., Wang, P., Peng, M., Shao, X., Chen, X. L., and Li, C. Y. (2018) Crystal structure of the dimethylsulfide monooxygenase DmoA from *Hyphomicrobium sulfonivorans*. *Acta Crystallographica Section F: Structural Biology Communications* **74**, 781-786

14. Fisher, A. J., Thompson, T. B., Thoden, J. B., Baldwin, T. O., and Rayment, I. (1996) The 1.5-Å resolution crystal structure of bacterial luciferase in low salt conditions. *J. Biol. Chem.* **271**, 21956-21968

15. Campbell, Z. T., Weichsel, A., Montfort, W. R., and Baldwin, T. O. (2009) Crystal structure of the bacterial luciferase/flavin complex provides insight into the function of the β subunit. *Biochemistry* **48**, 6085-6094

16. Nguyen, Q. T., Trinco, G., Binda, C., Mattevi, A., and Fraaije, M. W. (2017) Discovery and characterization of an F_{420}-dependent glucose-6-phosphate dehydrogenase (Rh-FGD1) from *Rhodococcus jostii* RHA1. *Appl. Microbiol. Biotechnol.* **101**, 2831-2842

17. Kertesz, M. A., Schmidt-Larbig, K., and Wuest, T. (1999) A novel reduced flavin mononucleotide-dependent methanesulfonate sulfonatase encoded by the sulfur-regulated *msu* operon of *Pseudomonas aeruginosa*. *J. Bacteriol.* **181**, 1464-1473

18. Eichhorn, E., van der Ploeg, J. R., and Leisinger, T. (1999) Characterization of a two-component alkanesulfonate monooxygenase from *Escherichia coli*. *J. Biol. Chem.* **274**, 26639-26646
